# Oncogenic CALR mutant C-terminus mediates dual binding to the thrombopoietin receptor triggering complex dimerization and activation

**DOI:** 10.1038/s41467-023-37277-3

**Published:** 2023-04-05

**Authors:** Nicolas Papadopoulos, Audrey Nédélec, Allison Derenne, Teodor Asvadur Şulea, Christian Pecquet, Ilyas Chachoua, Gaëlle Vertenoeil, Thomas Tilmant, Andrei-Jose Petrescu, Gabriel Mazzucchelli, Bogdan I. Iorga, Didier Vertommen, Stefan N. Constantinescu

**Affiliations:** 1 Ludwig Institute for Cancer Research Brussels, Brussels, Belgium; 2 Université catholique de Louvain and de Duve Institute, Brussels, Belgium; 3 Spectralys Biotech SRL, rue Auguste Piccard 48, 6041 Gosselies, Belgium; 4 Department of Bioinformatics and Structural Biochemistry, Institute of Biochemistry of the Romanian Academy, Splaiul Independentei 296, Bucharest, 060031 Romania; 5 Department of Molecular Biology and Genetics, Bilkent University, Ankara, Turkey; 6 Mass Spectrometry Laboratory, MolSys Research Unit, Universiy of Liège, 4000 Liège, Belgium; 7 Université Paris-Saclay, CNRS, Institut de Chimie des Substances Naturelles, UPR 2301 Gif-sur-Yvette, France; 8 de Duve Institute and MASSPROT platform, Brussels, Belgium; 9 Walloon Excelence in Life Sciences and Biotechnology, WELBIO, avenue Pasteur, 6, 1300 Wavre, Belgium; 10 Ludwig Institute for Cancer Research, Nuffield Department of Medicine, Oxford University, Oxford, UK

**Keywords:** Myeloproliferative disease, Structural biology

## Abstract

Calreticulin (CALR) frameshift mutations represent the second cause of myeloproliferative neoplasms (MPN). In healthy cells, CALR transiently and non-specifically interacts with immature N-glycosylated proteins through its N-terminal domain. Conversely, CALR frameshift mutants turn into rogue cytokines by stably and specifically interacting with the Thrombopoietin Receptor (TpoR), inducing its constitutive activation. Here, we identify the basis of the acquired specificity of CALR mutants for TpoR and define the mechanisms by which complex formation triggers TpoR dimerization and activation. Our work reveals that CALR mutant C-terminus unmasks CALR N-terminal domain, rendering it more accessible to bind immature N-glycans on TpoR. We further find that the basic mutant C-terminus is partially α-helical and define how its α-helical segment concomitantly binds acidic patches of TpoR extracellular domain and induces dimerization of both CALR mutant and TpoR. Finally, we propose a model of the tetrameric TpoR-CALR mutant complex and identify potentially targetable sites.

## Abstract (web summary)

In myeloproliferative neoplasms, frameshift mutants of calreticulin turn into rogue cytokines by inducing constitutive activation of the Thrombopoietin Receptor (TpoR). Here, the authors define how mutant calreticulin acquires specificity for TpoR binding and triggers its constitutive activation.

## Introduction

Myeloproliferative neoplasms (MPNs) are blood malignancies driven by the acquisition of somatic mutations in hematopoietic stem cells^1^. Frameshift mutations in the endoplasmic reticulum (ER) resident chaperone calreticulin (CALR) are the main cause of JAK2^V617F^ negative MPNs and are responsible for ~25% of Essential Thrombocythemia (ET) and myelofibrosis cases^2,3^. The most common CALR frameshift mutations in MPN are a 52-bp deletion denoted CALR del52 (type 1) and a 5-bp insertion called CALR ins5 (type 2), but all referenced mutations lead to the replacement of the wild-type C-terminus and KDEL ER-retention motif by a new sequence rich in methionine and positively charged residues^2,3^. These CALR mutants acquire the ability to specifically bind and activate the thrombopoietin receptor (TpoR), resulting in constitutive activation of the JAK-STAT pathway^4–8^. The binding of CALR mutants to TpoR relies notably on the interaction between CALR N-domain and immature N-glycans on TpoR^8,9^. Yet, this type of interaction is not specific to TpoR as wild-type CALR naturally associates via its N-domain with thousands of immature N-glycosylated proteins in a cycle of attachment and liberation that ends once the protein achieves proper folding^10^. In addition, the deletion of CALR mutant C-terminus results in reduced binding to TpoR and loss of activation of the JAK-STAT pathway^4,11,12^. These observations suggest that the interaction between mutant CALR and TpoR derives from novel properties acquired by CALR frameshift mutants that go beyond the canonical interaction between CALR N-domain and immature N-glycosylated proteins^10^. In this study, we used a multidisciplinary approach to uncover the basis for the specific and stable interaction between CALR mutants and TpoR and unveil how this interaction leads to productive dimerization and activation of the TpoR. Understanding how frameshift mutations in a master chaperone result in novel binding capacities is of deep interest both conceptually and therapeutically as the detailed characterization of binding and activation mechanisms is required for the development of therapeutic inhibitors. This study further provides a complete model of the CALR mutant-TpoR complex, paving the way for the development of therapeutic avenues.

## Results

### Frameshift mutations in CALR C-terminus unmask its N-glycan binding domain

**Fig. 1:**
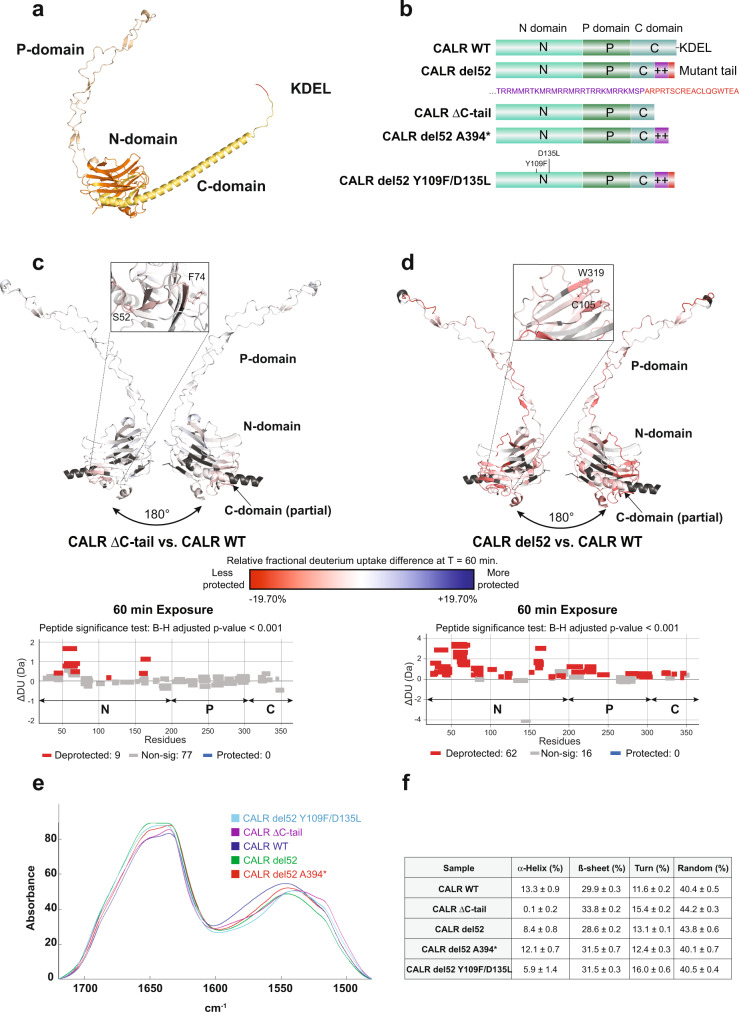
Structural changes induced by CALR frameshift mutation. **a** Structure of full length CALR WT predicted using AlphaFold 2.0^18^. The N-domain is shown in orange, the P-domain in wheat, the C-domain in yellow and the KDEL in red. **b** Representation of the domains and C-terminal sequences of CALR WT and CALR del52 or variants thereof used in FTIR spectroscopy and HDx-MS experiments. **c**, **d** Top: Structure model (AlphaFold 2.0^18^) of the common region between CALR WT and CALR del52 (corresponding to CALR ΔC-tail). Colors represent the difference in relative fractional uptake (ΔRFU) between CALR ΔC-tail and CALR WT (**c**) or between CALR del52 and CALR WT (**d**) at 1 h incubation in deuterium. Regions in red and blue are respectively less and more protected in CALR ΔC-tail (**c**) or CALR del52 (**d**) compared to CALR WT. The scale from red to blue is proportional to the ΔRFU between indicated CALR species with dark red and dark blue corresponding to highest differential. Dark gray represents regions without peptide coverage for the given time point. The N-glycans binding domain of CALR which is more exposed in CALR del52 compared to CALR WT is highlighted. Raw data are provided in the source file. Bottom: Wood’s plots generated with Deuteros 2.0^35^. Each bar (wood) represents the H-D exchange differential for a single peptide between indicated CALR species at 1 h incubation in deuterium. Peptides in red (deprotected) or blue (protected) have significant differential H-D exchange (*p* < 0.001) with the peptide-level significance testing as described^35^ (*n* = 3). The N-, P- and C-domains of CALR are indicated on the plots by letters N, P and C, respectively. Source data are provided as a Source data file. **e** Comparison of the mean spectra recorded for each sample to analyze the protein secondary structure. These spectra have been baseline-corrected and normalized. Each sample is identified by a unique color indicated in the legend. The unprocessed spectra are provided in Supplementary Fig. 4a. **f** Secondary structure predictions using the method developed on our in-house database (see Supplementary Methods). The prediction is realized on each individual FTIR spectrum. The average and the standard deviation for the 5 spectra recorded for each sample is shown in this table (*n* = 5). For the present predictions, the standard error of prediction in cross-validation is 5.7% for the *α*-helix and 6.7% for the ß-sheet, 3.2% for turns and 8% for random coil.

The stability of the interaction between CALR N-domain and immature N-glycans on TpoR^4,5,8,11^, our observation that CALR del52 exhibits a lower thermal stability than CALR WT^13^ (see also Supplementary Fig. 1a) and the fact that both CALR wild-type N- and mutant C-domains are required for TpoR binding^4,11,12^ together suggest that CALR frameshift mutations affect the structure of the whole protein. We therefore sought to compare the conformational footprints of CALR WT and mutants using hydrogen-deuterium exchange mass spectrometry (HDx-MS), a technique which uses mass shifts in peptides from a protein after hydrogen-deuterium (H-D) exchange in backbone amide positions to provide a readout of residue accessibility and protein conformation. CALR del52 (type 1) was chosen as the representative of CALR mutants that all acquire a very similar novel C-terminus^2,3^. To delineate the effect of the mutant C-terminus, we created CALR ∆C-tail. This variant contains the complete N-domain (residues 18–197), the proline-rich P-domain (residues 198–308) and part of the C-domain (residues 309–366) but not the C-terminal fragment (C-tail) that differs between CALR WT and CALR del52 (Fig. 1a, b). Similar deletions are frequent in a variety of solid-tumor cancers and are associated with immunosuppressive activity^14^. The three CALR variants (CALR WT, CALR del52 and CALR ∆C-tail) were produced as recombinant proteins and their purity and proper folding were validated by thermal shift, Coomassie blue staining and chromatography (Supplementary Fig. 1a, b; Supplementary Fig. 2). The HDx-MS footprints of CALR WT and variants were acquired with a sequence coverage of 92.6% (Supplementary Fig. 3a, b). Our analysis revealed that the absence of the last 50 amino acids of CALR (as in CALR ∆C-tail) did not drastically alter the conformation and accessibility of the rest of the protein. Only a small fragment of CALR N-domain was less protected in CALR ∆C-tail compared to CALR WT (Fig. 1c; Supplementary Fig. 3c). In sharp contrast, CALR del52 exhibited a globally more accessible conformation, except for fragments of the P-domain that were less accessible after 0.25 min incubation in deuterium but not at longer time points (Fig. 1d; Supplementary Fig. 3d). Critically, the N-domain of CALR del52 was the most affected by the addition of the mutant C-terminus and displayed a strong increase in H-D exchange compared to CALR WT. This sharp increase in accessibility was notably observed in residues involved in direct interactions with immature N-glycans such as C105 and W319^8,15^ (Fig. 1d), indicating that the region involved in binding immature N-glycans is unmasked due to the presence of the CALR del52 C-terminus.

### CALR mutant C-terminus contains two segments with distinct secondary structure

Next, we used Fourier transform infrared spectroscopy (FTIR) to define how the frameshift mutation in CALR del52 influences its secondary structure (Supplementary Figs. 4 and 5). Analysis of the amide I vibration revealed that the protein has high ß-sheet and random coil content, which is abundant in the N- and P-domains^16,17^ respectively, and that α-helical content was higher in CALR WT (13.3%) compared with CALR del52 (8.4%) (*p* < 0.001). The FTIR spectra of CALR ∆C-tail revealed that deletion of the last 50 residues resulted in an almost complete loss of α-helical content (0.1%), indicating that helicity is concentrated in the C-terminus of both wild-type and mutant CALR (Fig. 1e, f). The C-terminus of CALR mutant can be further separated in two segments based on amino acid composition. The proximal segment is rich in hydrophobic (Met) and basic (Arg, Lys) residues (Fig. 1b, purple) while the C-terminal segment starting at A394 (Fig. 1b, red) has a more heterogenous amino acid composition. FTIR spectra indicated that deletion of the last 18 residues (as in CALR del52 A394*) resulted in relative increase in α-helix content (12.1%), revealing that helicity was concentrated in the proximal segment of the mutant C-terminus rich in Arg and Met, in line with in silico prediction^18^ (Supplementary Fig. 1d). Because ß-sheets are concentrated in the N-domain^17^ (Fig. 1a) and α-helices present only in the C-terminus, these changes coupled to our HDx-MS data indicate that the acquisition of frameshift mutations in the C-terminus of CALR del52 disturbs its secondary structure and increases accessibility of CALR del52 N-domain.

Remarkably, introduction of two point mutations, (Y109F/D135L) in the N-domain of CALR del52 that abolish immature N-glycan binding^8,15^ also led to decreased helicity (from 8.4 to 5.9%) and of random coil percentage with a compensatory increase in turns and ß-sheets (Fig. 1e, f), suggesting that the different domains of CALR are conformationally linked.

### CALR mutant C-terminus directly interacts with a mature form of TpoR extracellular domain

**Fig. 2:**
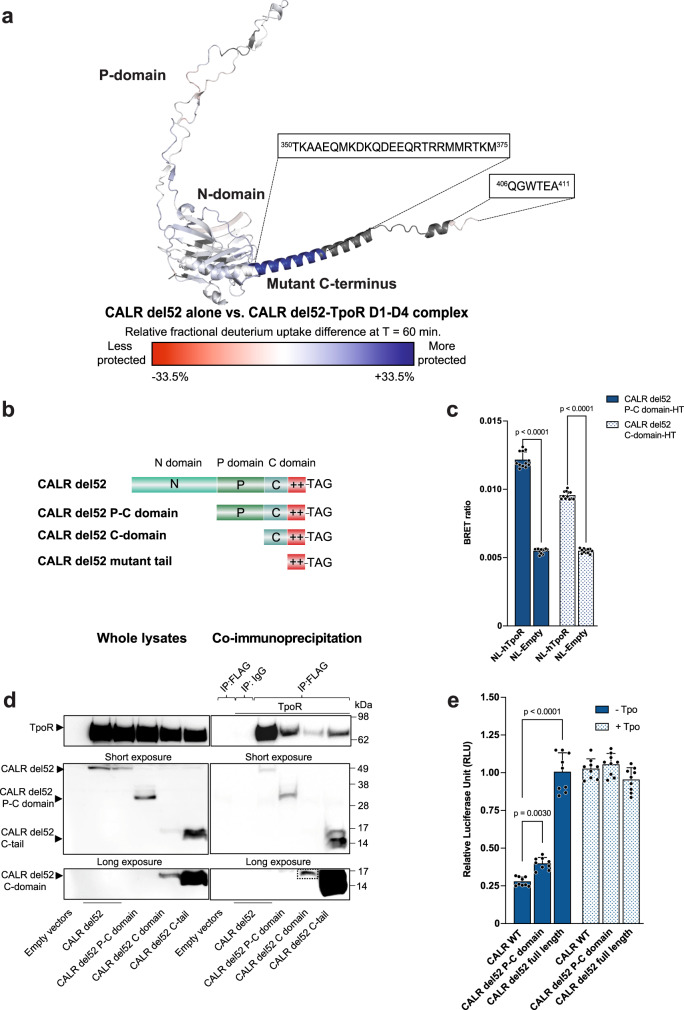
Interaction between CALR mutant C-terminus and TpoR independently of N-glycans. **a** Structure model (AlphaFold 2.0^18^) of CALR del52. Colors represent the difference in relative fractional uptake (ΔRFU) between CALR del52 alone and CALR del52 in complex with TpoR D1-D4 with mature N-glycans. Regions in red and blue are respectively less and more protected in the CALR del52-TpoR D1-D4 complex compared to CALR del52 alone. The scale from red to blue is proportional to the ΔRFU at 1 h incubation in deuterium between indicated species (mean of *n* = 3 experiments), with dark red and dark blue corresponding to highest differential. Dark gray represents regions without peptide coverage for the given time point. Sequences of the CALR mutant C-terminus are highlighted. Source data are provided as a Source data file. **b** Representation of N-terminal truncations of CALR del52 fused to either a FLAG tag or a HaloTag at the C-terminus. **c** NanoBRET between NanoLuc-TpoR (NL-TpoR) or NanoLuc not fused to any protein (NL-Empty) and CALR del52 P-C or C-domain-HaloTag. Data represent mean ± SD (*n* = 12 biologically independent samples over 4 independent experiments). Data were analyzed by two-ways ANOVA followed by Sidak multiple comparison test. Source data are provided as a Source data file. **d** Representative co-immunoprecipitation (from 3 independent experiments) of HA-TpoR with CALR del52-FLAG full length or N-terminal truncations as indicated. Source data are provided as a Source data file. **e** STAT5 transcriptional activity induced by indicated CALR truncations in presence of TpoR. HEK293T were transiently transfected with vectors coding for human TpoR and CALR del52 truncations along with cDNAs coding for STAT5, JAK2 and SpiLuc Firefly luciferase reporter reflecting STAT5 transcriptional activity and normalized with a control reporter (pRLTK) containing Renilla luciferase. Data represent mean ± SD (*n* = 9 biologically independent samples over 3 independent experiments). Data were analyzed with two-ways ANOVA followed by Sidak multiple comparison test. Source data are provided as a Source data file.

Although the higher accessibility of CALR del52 N-domain could explain its capacity to form a stable interaction with immature N-glycans of TpoR, the specificity of CALR mutant for TpoR versus other cytokine receptors^8^ suggested that other binding mechanisms could be at play. To probe this assumption, we produced and purified recombinant TpoR extracellular domain (ECD) labeled TpoR D1-D4 (Supplementary Figs. 2g and 6a, b) that contains mature N-glycans^8^ to avoid any generic interaction between CALR N-domain and immature N-glycans. The binding affinity between the mature TpoR ECD and CALR del52 was evaluated using microscale thermophoresis to ~104 nM (Supplementary Fig. 6c). Then, we set up a HDx-MS experiment to determine which regions of CALR del52 interacted with TpoR ECD in absence of immature N-glycans. Considering the level of affinity between the two partners, the proteins were incubated at 1:1 molar ratio (at 20 μM each) prior to experiments. At this concentration, the percentage of complex at steady state is of ~93% given a ~104 nM affinity. The HDx-MS footprint of CALR del52 was acquired with a sequence coverage of 89.6% (Supplementary Fig. 6d). Comparison of the H-D exchange between CALR del52 alone or in presence of TpoR ECD revealed significant (*p* < 0.01) H-D exchange differential in different peptides containing CALR del52 C-terminus (Fig. 2a and Supplementary Fig. 6e, f), indicative of a direct interaction with the mature TpoR ECD. This differential exchange was not observed in the C-terminal extremity of the mutant C-terminus encompassing residues ^406^QGWTEA^411^ (Supplementary Fig. 6e, f) but only in the α-helical segment containing positively charged residues. Fragments of the N-domain predicted to be conformationally close to the C-domain also exhibited significant, albeit smaller, decreased accessibility in presence of the mature TpoR ECD (Supplementary Fig. 6e). Thus, CALR mutant C-terminus interacts with TpoR in the absence of the interaction between the N-terminus of CALR del52 and immature N-glycans. Importantly, in this work we show later that the interaction is maintained also when the N-domain interacts with immature N-glycans.

To validate this interaction in living cells, we used Nano-bioluminescence energy transfer (NanoBRET) where close proximity (<10 nm) between a bioluminescent energy donor (NanoLuc) and a fluorescent energy acceptor (HaloTag) results in energy transfer measured by the BRET ratio. We measured the BRET ratios between fragments of CALR del52-HaloTag and NanoLuc-TpoR as we did before for full length CALR del52^8^. We deleted the N-domain or both N- and P-domains of CALR del52 to create the constructs labeled P-C and C-domain, respectively (Fig. 2b), which do not contain the N-domain required to bind immature N-glycans^15,19^. Both truncated forms of CALR del52 retained significant interaction with TpoR in living cells as measured by the BRET ratio (Fig. 2c; Supplementary Fig. 7). This interaction was further validated by co-immunoprecipitation between FLAG-tagged N-terminal truncations of CALR del52 and HA-tagged TpoR (Fig. 2d). Then, we questioned whether CALR del52 devoid of N-glycan binding domain remained able to induce TpoR activation. We used a luciferase assay^20^ to measure the STAT5-dependent transcriptional activity in cells co-expressing TpoR and CALR del52 P-C domain. Remarkably, the latter conserved the ability to induce a significant induction of STAT5 transcriptional activity in presence of TpoR (Fig. 2e) but not of the erythropoietin receptor (EpoR) (Supplementary Fig. 6g), indicating that the deletion of CALR mutant N-domain does not completely inhibit its ability to specifically bind and activate TpoR.

### Mapping of interactions in the TpoR-CALR mutant complex

**Fig. 3:**
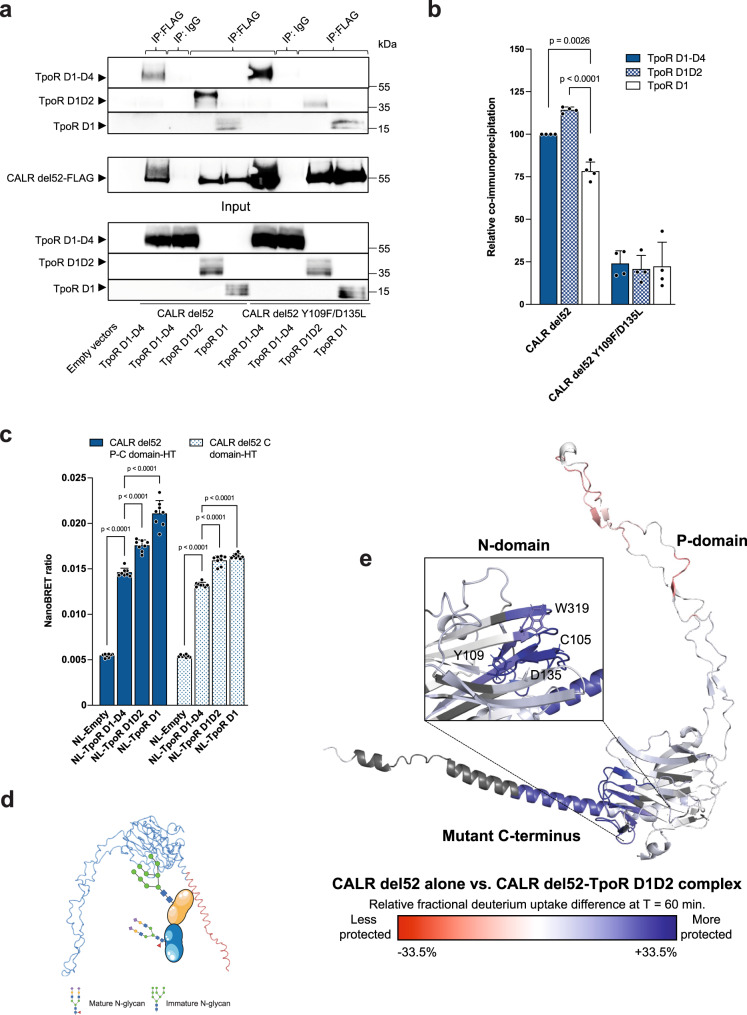
N-glycan dependent and independent interactions of CALR mutant with TpoR. **a** Representative co-immunoprecipitation of HA-TpoR ECD domains by CALR del52-FLAG or CALR del52 Y109F/D135L-FLAG using an anti-FLAG antibody for capture and anti-HA antibody or anti-CALR mutant C-terminus antibody (SAT602) for detection of HA-TpoR and CALR del52-FLAG (and Y109F/D135L mutant), respectively. Source data is provided as a Source data file. **b** Quantification of relative co-immunoprecipitation of TpoR species by CALR del52 (mutated or not). Western blot quantification performed with ImageJ. Shown are the ratios (+ SD) of TpoR species on CALR del52 normalized for TpoR species expression in whole lysates (*n* = 4). Data were analyzed by two-ways ANOVA followed by SIDAK multiple comparison test. Source data are provided as a Source data file. **c** NanoBRET between NanoLuc-TpoR subdomains (or NL-Empty) and CALR del52-HaloTag truncated from the N-terminus. Data represent mean + SD (*n* = 8 biologically independent samples from 4 independent experiments). Source data are provided as a Source data file. **d** Cartoon representing the complex between CALR del52 and TpoR D1D2 domain containing immature N-glycans produced in Schneider (S2) cells. Items in this figure were created with BioRender. **e** Structure model (AlphaFold 2.0^18^) of CALR del52. Colors represent the difference in relative fractional uptake (ΔRFU) at 1 h incubation in deuterium (mean of *n* = 3 experiments) between CALR del52 alone and CALR del52-TpoR D1D2 complex with immature N-glycans. Regions in red and blue are respectively less and more protected in the CALR del52-TpoR D1D2 complex compared to CALR del52 alone. The scale from red to blue is proportional to the ΔRFU between indicated species with dark red and dark blue corresponding to highest differential. The same scale as Fig. 2a was used for comparison. Raw data are provided in the source file. Dark gray represents regions without peptide coverage for the given time point. The N-glycan binding domain of CALR is highlighted. Source data are provided as a Source data file.

Having established that the C-terminus of CALR mutant directly interacts with TpoR ECD, we sought to identify the region of TpoR involved in this interaction. The TpoR ECD is composed of four subdomains labeled from D1 to D4 starting from the N-terminus. To determine which of these subdomains is involved in binding to CALR mutant, we first used co-immunoprecipitation between CALR del52 and progressive truncations of TpoR ECD starting from the C-terminal D4 fragment. In these conditions where TpoR fragments and CALR del52 are expressed in the same cell, TpoR fragments retain immature N-glycans^8^. Deletions of the D3D4 domains of TpoR did not reduce interaction with CALR del52 while deletion of D2 resulted in a modest decrease in co-immunoprecipitation compared to D1 alone (Fig. 3a, b), indicating that binding of TpoR to CALR del52 occurs essentially via the D1 domain. This is reminiscent of our previous finding that immature N-glycans attached to Asn117 of D1 are the major site of CALR del52 binding via the N-domain^5,8^. Given our result that CALR mutant C-terminus binds TpoR also in presence of mature N-glycans (Fig. 2), we additionally probed binding of TpoR ECD fragments to the CALR del52 Y109F/D135L double mutant that is deficient for binding immature N-glycans^8,15^. Expectedly, loss of N-glycan-dependent interaction led to a sharp decrease in co-immunoprecipitation ratios (Fig. 3b). However, unlike with non-mutated CALR del52, the interaction of CALR del52 Y109F/D135L was similar between D1 and D1D2 ECD fragments (Fig. 3a, b), suggesting that interaction between CALR mutant C-terminus and TpoR occurs essentially via the D1 domain. Noteworthy, the TpoR D1D2 species that co-immunoprecipitated with CALR del52 Y109F/D135L had a smaller apparent molecular size than that interacting with non-mutated CALR del52 (Fig. 3a). This small size shift, visible only for TpoR D1D2 due to better resolution in this part of the gel, correlates with the fact that the TpoR D1D2 that interacts with non-mutated CALR del52 retains immature N-glycans^8^, unlike the TpoR D1D2 mature species that binds the CALR del52 Y109F/D135L double mutant that is deficient for N-glycan binding. To confirm this in live cells, we used our NanoBRET assay as in Fig. 2c to measure binding between CALR del52 P-C or C-domain-HaloTag and fragments of NanoLuc-TpoR ECD. Confirming results from co-immunoprecipitations, the interaction between all fragments of TpoR ECD and CALR del52 P-C and C-domain was conserved (Fig. 3c). Strikingly, the interaction between CALR del52 P-C or C-domain and TpoR ECD was even increased in D1 and D1D2 compared to the full TpoR ECD, possibly indicating that removing the C-terminal segments of TpoR ECD improves accessibility of the D1 domain to CALR mutant C-terminus.

### CALR mutant interacts with TpoR via two major domains

In the physiologically relevant CALR mutant-TpoR complex, TpoR retains immature N-glycans attached to Asn117^8^. We thus sought to assess whether the presence of immature N-glycans on TpoR affected the H-D exchange profile of CALR del52 in presence of TpoR ECD and to identify the binding sites of CALR del52 with immature N-glycans on TpoR. The recombinant CALR del52-TpoR D1D2 complex was produced in S2 cells, and its purity was verified by thermal shift assay, Coomassie blue staining and size-exclusion chromatography (Fig. 3d; Supplementary Figs. 2f and 8a, b). In this complex, immature N-glycans are attached to Asn117^8^. Comparison of the H-D exchange profile between CALR del52 alone or in complex with TpoR D1D2 with immature N-glycans (sequence coverage of ~90%, Supplementary Fig. 8c) indicated that CALR del52 interacted with TpoR via two major domains. The strongest H-D differential was observed in the putative N-glycans binding site of CALR (Fig. 3e; Supplementary Fig. 8d). This region included notably C105, Y109, D315 and W319 that were reported to be key for binding of immature N-glycans^8,19,21,22^ and were all more protected in presence of TpoR. Importantly, this region is not involved in binding to mature TpoR (Fig. 2a), indicating that these residues are specifically involved in the interaction with immature N-glycans. The second major H-D differential was present in peptides containing the mutant C-terminus of CALR del52 which exhibited strong protection in the TpoR-CALR mutant complex compared to CALR mutant alone (Fig. 3e; Supplementary Fig. 8d, e). These peptides were similar to the ones exhibiting differential H-D uptake between CALR del52 alone or in complex with mature TpoR (Fig. 2; Supplementary Fig. 6e, f), indicating that CALR mutant C-terminus interacts with both mature and immature forms of TpoR ECD. Interestingly, fragments of the P-domain were significantly more accessible in the CALR mutant-TpoR complex (Figs. 2a, 3e and Supplementary Figs. 6e, 8d). This observation suggests that in absence of TpoR, the P-domain may interact with CALR N- or C-domain and that this interaction is destabilized upon binding to TpoR. This hypothesis is also supported by previous reports that the deletion of CALR mutant P-domain improves the binding of CALR mutant to TpoR^4^.

### CALR mutant C-terminus interacts with acidic patches on TpoR D1 domain

**Fig. 4:**
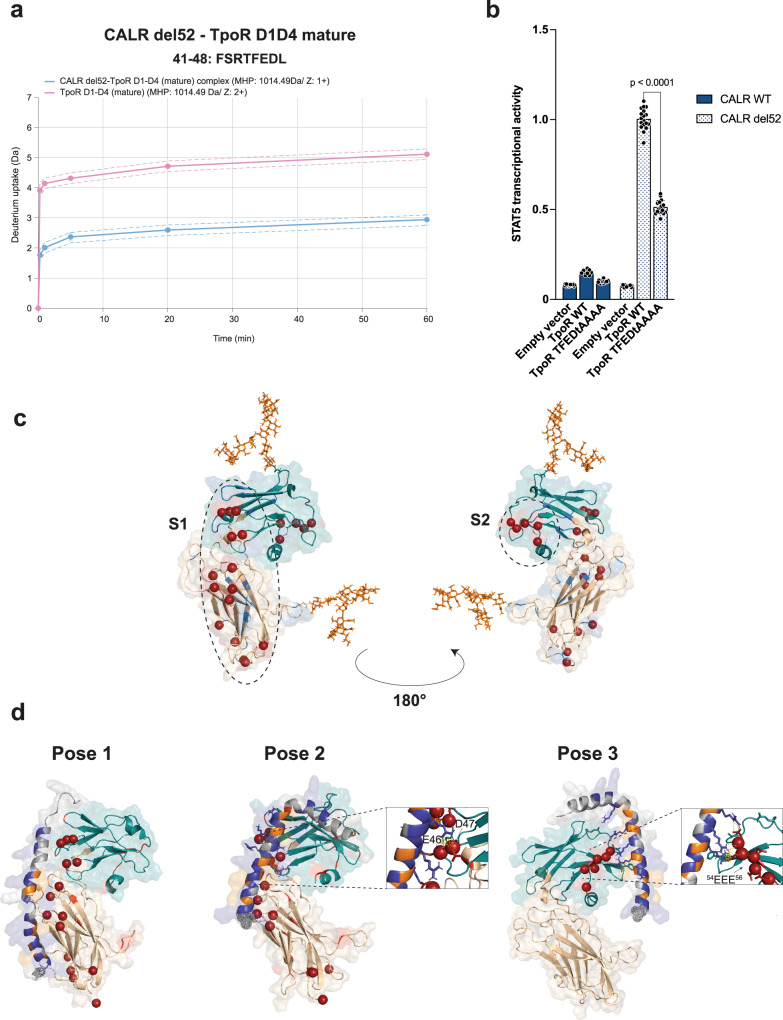
CALR mutant C-terminus interacts with acidic patches on TpoR D1 domain. **a** Deuterium uptake (Da) of the FSRTFEDL peptide from CALR del52 alone or in complex with TpoR D1D4 (with mature N-glycans) at 5 different exchange time points. The dotted lines represent standard deviation (SD), the full line represents the mean of triplicates (*n* = 3). Source data are provided as a Source data file. **b** STAT5 transcriptional activity induced by CALR del52 in presence of empty vector, TpoR WT or TFEDtAAAA mutant. Data represent mean ± SD (*n* = 12 biologically independent samples over 4 independent experiments). Data were analyzed with one-way ANOVA followed by Sidak multiple comparison test. Source data are provided as a Source data file. **c** Prediction of TpoR D1D2 domain (described in Supplementary Methods). The model shows one extensive (S1) and one more restricted (S2) patch of acidic residues represented by red spheres. Basic residues are shown in blue, acidic residues are shown in red. TpoR D1 domain is shown in blue/green and TpoR D2 is shown in wheat. Complex N-glycans are attached to position Asn117 and Asn178 and are shown in orange. **d** Pose 1 (left), pose 2 (middle) and pose 3 (right) generated by HADDOCK between CALR del52 mutant C-terminus and TpoR D1D2. The best docking complexes were chosen, as ranked by the HADDOCK score. Highlighted are the strong interactions between Arg of CALR mutant C-terminus and E46 and D47 of TpoR D1 domain for pose 2 and the interactions between Arg of CALR mutant C-terminus and the ^54^EEE^56^ motif on TpoR D1 for pose 3. The basic (Arg/Lys) and hydrophobic (Met) residues of CALR del52 mutant C-terminus are shown in dark blue and orange, respectively. Other residues are shown in gray.

To study with more precision the residues of TpoR ECD that may interact with CALR mutant C-terminus, we used HDx-MS with the same set-up as in Fig. 2a, where we showed that CALR del52 interacts with mature TpoR exclusively through the mutant C-terminus. H-D exchange on TpoR ECD was acquired with a lower sequence coverage (~50%) than for CALR del52 due to the presence of 4 N-glycans on TpoR ECD that complicated pepsin digestion (Supplementary Fig. 9a). Amongst the covered region, by far the strongest interaction was observed with the ^41^FSRTFEDL^48^ motif of TpoR S1 region (Fig. 4a), for which differential H-D uptake between TpoR ECD alone or in presence of CALR del52 was significant (*p* < 0.001) for all incubation time points (Fig. 4a; Supplementary Fig. 9b). Remarkably, the same peptide of TpoR ECD also exhibited strong differential H-D uptake when compared to the CALR del52-TpoR produced as a complex with immature N-glycans (Supplementary Fig. 9c). Consistently, mutations of the ^44^TFED^47^ motif to alanine prevented TpoR activation by CALR del52 in our STAT5 transcriptional luciferase assay (Fig. 4b). In addition, lower but significant differential H-D exchange was also detected for the ^52^WDEEEAAPSGT^62^ peptide (Supplementary Fig. 9d).

Next, we turned to molecular dynamics (MD) simulations to study the configurations in which CALR mutant C-terminus could interact with TpoR ECD. We first generated the structure of TpoR D1D2 and CALR del52 C-terminus (Fig. 4c; Supplementary Fig. 10a). Based on previous characterization of the TpoR N-glycans composition in the TpoR D1D2-CALR del52 complex^8^, immature (high-mannose) and mature N-glycans were attached to Asn117 and Asn178 of TpoR, respectively. The final model was similar to the one generated with AlphaFold 2.0^18^. Sequence analysis indicated that TpoR D1D2 exhibits an unbalanced charge composition with an excess of 11 negatively charged acidic amino acids with one extensive (S1) and a second more localized (S2) negatively charged region (Fig. 4c). Since CALR mutant C-terminus is strongly positively charged and that the two peptides identified by HDx-MS are rich in negatively charged residues, we hypothesized that electrostatic interactions could mediate binding between CALR mutant C-terminus and TpoR ECD. To challenge this assumption, we generated mutants of CALR del52 where all hydrophobic (Met) or basic amino acids (Arg/Lys) of the mutant C-terminus are replaced by either Gly or Asn residues. Using our STAT5 transcriptional assay, we observed that mutations of basic but not of hydrophobic residues to either Gly or Asn resulted in complete loss of TpoR activation by CALR del52 (Supplementary Fig. 9e, f), in line with previous reports^12^. Taking into consideration the localization of the negatively charged acidic residues on TpoR, three main start configurations (poses) of the complex were chosen for assessing complex formation and were used as inputs in HADDOCK 2.4^23^ for complex optimization searches. The poses predicted interaction either with the extended S1 region of TpoR (pose 1 and pose 2) or via the more restricted S2 region (pose 3) (Fig. 4d). Starting from the poses generated by HADDOCK (Supplementary Fig. 10b), each of the three poses were subjected to triplicate 500 ns unconstrained MD stimulations (Supplementary Fig. 10c–f). The interaction of CALR mutant C-terminus with the Asp and Glu residues of the ^44^TFED^47^ motif was consistent with this observed in silico with pose 2 (Fig. 4c, d) and this interaction was conserved after 500 ns of unconstrained MD simulations in all three replicates (Supplementary Fig. 11). Similarly, binding to the ^52^WDEE^55^ motif was compatible with pose 3 of our model (Fig. 4c).The free energy (ΔG) of the three poses was then estimated by both a knowledge-based method (using the PRODIGY server^24^) and a physical MD estimation (using the MM-GBSA method^25^) at 150 mM salt concentration (Supplementary Fig. 12 and Supplementary Methods). Both methods indicated that in all three poses CALR del52 displays a very high affinity for TpoR D1D2 (ΔG < −9 kcal/mol) with the same order of magnitude for poses P1, P2 and P3. Moreover, MD simulations identified in each pose multiple microstates of the complex (Supplementary Fig. 13 and Supplementary Methods) that can target both the continuous area found mainly on D1 (and partly on D2) labeled S1, but also the small acidic patch in the N-terminal region of D1 (S2). Taken together, these results suggest that, in absence of stabilization by immature N-glycans, multiple micro-configurations of CALR del52-TpoR ECD can co-exist but that the S2 patch centered on ^44^TFED^47^ plays a central role in binding.

### Dimerization of TpoR and of CALR del52 is mediated by the α-helical segment of CALR mutant C-terminus

Our data indicated that both CALR mutant C-terminus and N-domain are involved in direct interaction with TpoR. Previous studies provided evidence that CALR mutant C-terminus is also indispensable for activation of the receptor^4,12^ which occurs after homodimerization of mutant CALR^26^. Yet, exactly how activation is achieved remained unclear.

**Fig. 5:**
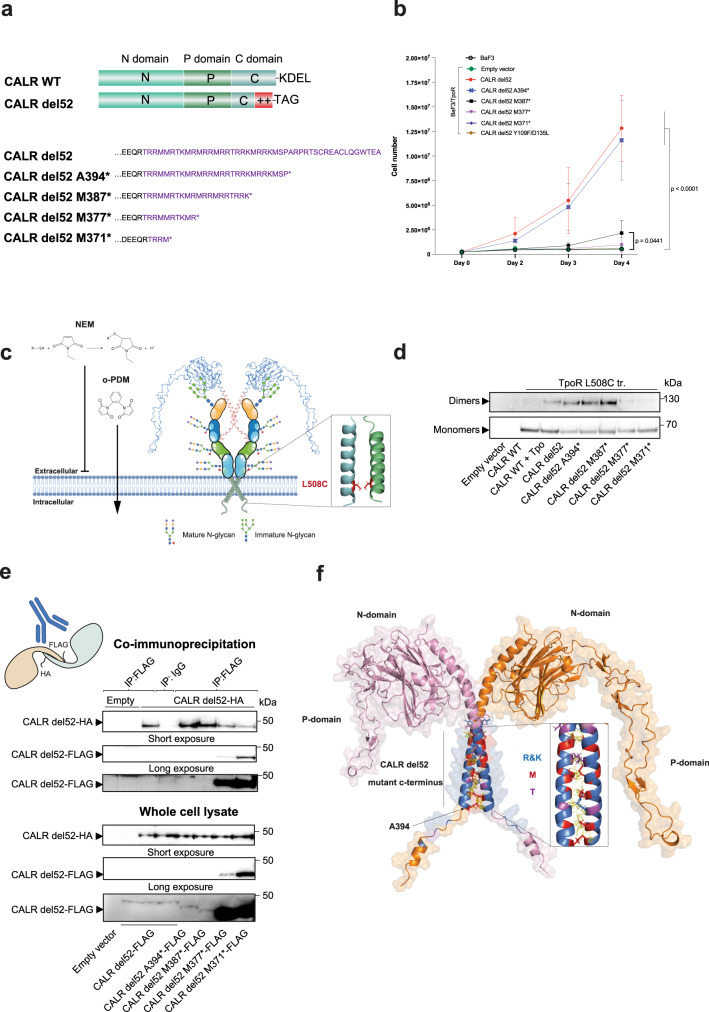
Dimerization of TpoR and of CALR del52 is mediated by the α-helical segment of CALR mutant C-terminus. **a** Representation and sequence of CALR del52 C-terminal truncations used in proliferation assay. **b** Proliferation assay. Ba/F3 cells stably expressing TpoR in pMX-IRES-GFP vector were transduced with indicated CALR variants or an empty vector (pMSCV-IRES-mCherry) and sorted by FACS. 250,000 cells were washed and seeded in 10 mL of complete culture medium without cytokine and counted each day using an automated cell counter. Values represent mean of 3 independent experiments (±SD) (*n* = 3). Source data are provided as a Source data file. Data were analyzed using two-ways ANOVA followed by Sidak multiple comparison test at day 4 using the average of technical triplicate counting from 3 independent experiments (*n* = 3). **c** Cartoon representation of the crosslinking assay to assess homodimerization of TpoR in a productive orientation. o-PDM: ortho-phenylene dimaleimide. NEM: N-ethylmaleimide. Items in this figure were created with BioRender. **d** Crosslinking study of TpoR dimerization in presence of Tpo, CALR del52 full length or C-terminal truncations. Shown is a representative western blot (from 3 independent experiments) in denaturing and reducing conditions showing human TpoR monomers and o-PDM crosslinked dimers in the indicated conditions. Source data are provided as a Source data file. **e** Co-immunoprecipitation of CALR del52-HA full length by CALR del52-FLAG full length or truncated to assess dimerization. Shown are representative western blots in denaturing conditions (from 3 independent experiments). Source data are provided as a Source data file. **f** RosettaDock top scoring simulation of CALR del52 dimers. Structure of CALR del52 was modeled using AlphaFold 2.0^18^. CALR del52 monomers are shown in orange and pink. Residues of the CALR mutant C-terminus are shown in purple (Thr), dark blue (Arg) and red (Met). Close interactions (<3Ä) are shown by yellow dashed lines.

To close this gap, we sought to determine the exact region of CALR mutant C-terminus required to induce TpoR activation and dimerization. We measured autonomous proliferation of cytokine-dependent hematopoietic cells (Ba/F3) stably expressing TpoR together with progressive truncations of the C-terminus of CALR del52 (Fig. 5a). The deletion of the non α-helical segment of CALR mutant C-terminus (as in CALR del52 A394*) (Fig. 1e, f) did not prevent TpoR-dependent proliferation of Ba/F3 cells. In contrast, further deletions in the α-helical segment of CALR del52 either reduced (M387*) or prevented (M377* and M371*) CALR del52 mediated activation of TpoR (Fig. 5b). Similarly, CALR del52 Y109F/D135L mutant, which is deficient for N-glycan binding and disturbs the helicity of the mutant C-terminus (Fig. 1e, f), was not able to induce Ba/F3 autonomous proliferation in presence of TpoR.

Since activation of homodimeric cytokine receptors occurs upon ligand-induced dimerization^27^ in a productive conformation^28,29^, we then assessed whether the same truncations of CALR del52 C-terminus precluded homodimerization of TpoR in a live-cell cysteine crosslinking assay. The L508C point mutation, homologous to murine L501C, was introduced in human TpoR, placing the cysteine residue at a position facing inward the transmembrane *α*-helix in the active conformation of TpoR dimer^28^. The specificity of the crosslinking was achieved by using a truncated form of the TpoR devoid of intracellular cysteines which remains active^28^ and by preventing crosslinking of free cysteines of the ECD by pre-incubation with N-ethyl-maleimide which blocks free extracellular cysteines (Fig. 5c). In agreement with above results, truncations until M387 allowed CALR del52 induced dimerization of TpoR while further truncations precluded the formations of homodimers (Fig. 5d).

Because oligomerization of CALR mutants themselves precedes TpoR activation^26,30^, the same set of CALR del52 C-terminal deletions (Fig. 5a) was used to probe the role of the α-helical segment of CALR mutant C-terminus in CALR homodimerization. By co-immunoprecipitating HA-tagged full length CALR del52 with FLAG-tagged CALR del52 truncations, we observed that truncations beyond the non α-helical segment strongly decreased CALR del52 oligomerization, indicating that the same 28 α-helical residues of the mutant C-terminus required for TpoR activation and dimerization also mediate CALR oligomerization (Fig. 5e). Consistently, the oligomeric profile in native conditions was similar between recombinant CALR del52 and CALR del52 A394* with or without reducing agent (indicating that C-terminal cysteines are not required for homo-multimerization) while CALR ΔC-tail did not form any oligomer (Supplementary Fig. 14).

Finally, we used RosettaDock^31^ to model CALR del52 dimer formation using the monomeric structure predicted using AlphaFold 2.0^18^. The top 10 models predicted dimerization through the mutant C-terminus via residues prior A394 and the two C-terminal cysteines. The best scoring prediction is depicted in Fig. 5f and shows dimerization of CALR del52 via the mutant C-terminus which forms a coiled-coil like structure with interactions involving Arg (dark blue), Met (orange) and Thr (purple), but not the cysteines at the extremity of CALR mutant C-terminus.

### Comprehensive model of the TpoR-CALR mutant complex

**Fig. 6:**
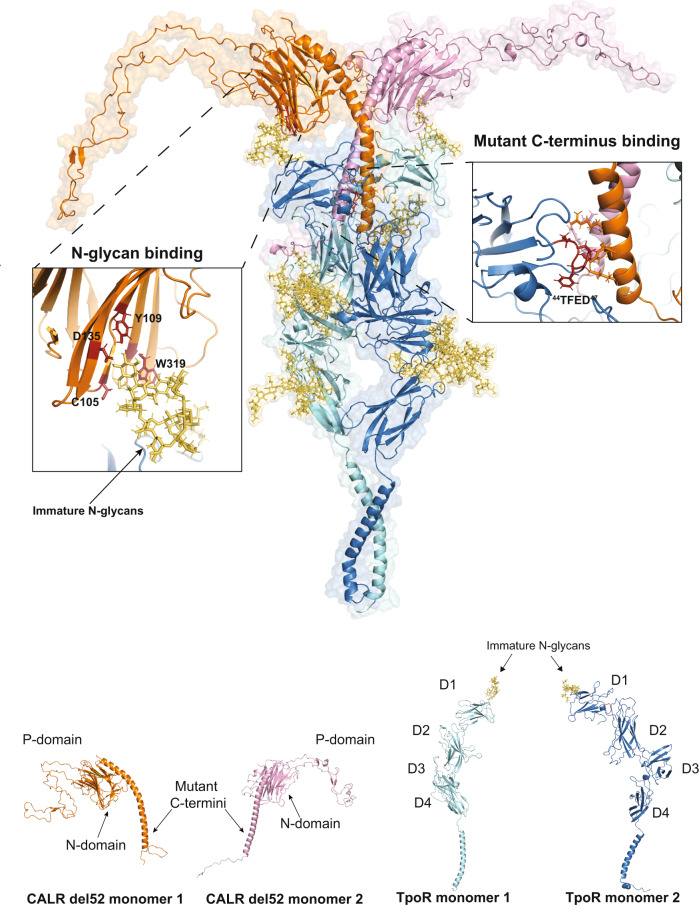
Comprehensive model of the TpoR-CALR mutant complex. Molecular dynamics of the CALR del52-TpoR tetrameric complex. The structure represents the last frame of one out of three replicates of 100 ns unconstrained MD of the CALR del52-TpoR tetrameric complex. All replicates are shown in Supplementary Fig. 15. The model illustrates how CALR mutant interacts with TpoR via two distinct regions. One region concerns the interaction between CALR N-domain (N-glycan binding domain) and immature N-glycans attached to Asn117 of TpoR. The second region involves binding of CALR mutant C-terminus to acidic patches on TpoR ECD. The interaction between TpoR negative residues and CALR mutant C-terminus may possibly occur both in *cis* (with the same molecule of CALR mutant with which TpoR interacts via N-glycans) or in *trans*. The binding between specific residues of CALR N-domain and immature N-glycans attached to Asn117 and that of the ^44^TFED^47^ motif of TpoR to CALR mutant C-termini is illustrated. The contact list of interactions detected during MD triplicate runs is provided as Supplementary data 1. TpoR molecules are shown in cyan and dark blue. CALR del52 molecules are shown in orange and pink. Key residues of CALR del52 N-domain involved in binding of immature N-glycans are shown in dark red. The ^44^TFED^47^ motif of TpoR D1 domain that interacts with CALR del52 C-termini is shown in red. The different domains of CALR mutant and TpoR are indicated.

On the basis of our experimental data, we generated an atomistic model of the complete TpoR-CALR mutant tetrameric complex. Our data indicated that binding of CALR del52 C-terminus alone to TpoR could occur in a variety of micro-configurations but that the extended S1 acidic region centered on ^44^TFED^47^ was key for interaction. Yet, binding of TpoR to full length CALR del52 also involves strong interaction between specific residues of the N-domain and immature N-glycans on Asn117^8^ (Fig. 3e). We used AlphaFold 2.0^18^ to complete our modeling of TpoR and generate the full extracellular domain and transmembrane domain of the receptor. TpoR monomers were dimerized through their TM domain with residue L508 in the interface as in the active configuration in presence of CALR del52 (Fig. 5c, d). CALR del52 dimer (Fig. 5f) was docked to the dimer of TpoR taking into consideration our experimental data indicating that binding occurs concomitantly between immature N-glycans on Asn117 of TpoR and residues of CALR N-domain and between TpoR S1 acidic region and CALR mutant C-terminus. The final structure places the mutant C-terminus in a configuration where the main interacting sites are located around the ^44^TFED^47^ motif, in line with above results. Likewise, immature N-glycans on Asn117 of TpoR interact with the N-domain pocket containing key residues involved in N-glycan binding including C105, Y109 and W319 (Fig. 6). This glycoproteic tetramer was embedded in a POPC lipid bilayer and a water box (comprising a total of ~1 million atoms) and subjected to triplicate all-atom molecular dynamics simulations for 100 ns. The complex remained stable during this timeframe, except for the very flexible P-domain (Supplementary Fig. 15). Most contacts identified during the simulations relied on basic-acidic interactions and occurred both in *cis* and in *trans*, thereby further stabilizing TpoR dimers. They involved the ^44^TFED^47^ motif but also other negative patches including ^96^PDQEE^100^ and ^154^WEEP^157^ of the extended S1 negative patch (Supplementary Data 1). To assess whether our model was also compatible with CALR ins5 (CALR type 2 mutant), which harbors a similar but longer C-terminus, we also generated the CALR ins5-TpoR tetrameric complex following the same procedure as for CALR del52 and subjected the complex to all-atom MD simulations in triplicate (Supplementary Fig. 16). Like for CALR del52, the complex remained stable over the 100 ns timeframe. Analysis of interacting residues over the simulation timeframe revealed that the ^44^TFED^47^ motif, ^96^PDQEE^100^ and ^154^WEEP^157^ motifs were conserved in the CALR ins5-TpoR tetrameric complex (Supplementary Data 2).

Our experimental data and atomistic simulations indicate that CALR mutants interact through two regions of TpoR essentially on the D1 domain. First, the mutant C-terminus directly interacts with multiple negatively charged residues on the inner/lateral face of TpoR D1 domain represented by the S1 negative patch. Given the ability of the mutant C-terminus to interact with multiple acidic residues on TpoR D1 domain, it is likely that different micro-configurations co-exist in living cells, especially in absence of immature glycans to stabilize one specific configuration. When this interaction occurs in the context of immature TpoR, strong interactions between CALR N-domain and immature N-glycans mainly on Asn117 of TpoR stabilize the complex. Thus, the mutant C-terminus provides the specificity and stability of the tetrameric complex.

## Discussion

Our work unveils the molecular basis for the recognition and activation of the thrombopoietin receptor by frameshift mutants of calreticulin that are at the origin of myeloproliferative neoplasm^2,3^. These findings provide mechanistic insights into the mechanisms leading to a switch from the transient, N-glycan-only based interaction between wild-type calreticulin and thousands of proteins to a specific and stable interaction between CALR frameshift mutants and TpoR. Our results indicate that this specificity relies on two complementary mechanisms. First, the presence of CALR mutant C-terminus induces a conformational overhaul of CALR N-domain, resulting in increased accessibility of the N-glycan binding pocket that interacts with immature N-glycans on Asn117 of TpoR^5,8^. Remarkably, this N-glycan binding pocket is reminiscent of the one that we and our collaborators recently identified as the hematoxylin binding site via which hematoxylin acts as an inhibitor of CALR del52 binding to TpoR^9^. Increased accessibility of this pocket in CALR del52 explains the partial specificity of hematoxylin to target mutant cells^9^. Secondly, our results demonstrate a direct interaction between the positively charged CALR mutant C-terminus and negatively charged residues on TpoR D1 domain. We posit that this second interaction is the basis for the specificity of CALR mutant for TpoR versus other N-glycosylated proteins. In addition, our HDx-MS results highlight several regions that exhibit different accessibility between CALR WT and CALR del52, suggesting potential targetable sites. Of interest, mutations in CALR C-terminus are also observed in a variety of non-hematological cancers and their exposure at the cell surface leads to immunosuppressive activity^14^. Conceptually, it is tempting to speculate that the enhanced accessibility of CALR N-terminus that our work identifies upon deletion of the C-terminus could be used for therapeutical targeting.

Most recent work in the field elegantly demonstrated using single molecule fluorescence tracking that TpoR is a monomer at the basal state and that dimerization is induced upon ligand binding to the ECD^27,32^. Several dimer conformations can be induced by synthesized ligands such as diabodies that can tune the receptor for subtly different effects that all require induced dimerization^32^. Previous studies demonstrated that CALR del52, like Tpo, can act as a cytokine to induce dimerization of TpoR^8,26^. Here, we define the basis for this pathological ligand-induced dimerization. We find that the first segment of CALR mutant C-terminus that is α-helical is involved in CALR oligomerization, TpoR binding and TpoR dimerization. This observation suggests that one face of the α-helix forms a coiled-coil like structure at the core of the CALR dimer while the other face interacts with negative patches of TpoR D1 domain (Fig. 6). These results contradict previous reports claiming that CALR mutant dimerization occurs through disulfide bonds involving cysteines of the C-terminal extremities of CALR mutant, and that the same cysteines of a fusion protein containing the mutant C-domain are involved in binding TpoR^33^. However, this was inferred from comparison of western blots in denaturing conditions with or without reducing agent, thus not reproducing the native conformation of the protein. While disulfide bonds may form after the Cys-independent interaction and activation, our live-cell cysteine crosslinking assays, oligomerization analysis in native conditions and co-immunoprecipitation studies all indicate that CALR mutant C-terminal cysteines are not required for CALR oligomerization nor for TpoR dimerization. Noteworthy, these results confirm our observation and those from others^11,12^ that C-terminal cysteines of CALR mutant are not involved in TpoR activation.

Finally, our experimentally validated in silico analyses identify specific residues of the TpoR D1 domain that interact with CALR mutant C-terminus. Identification of these interaction sites is of major importance for the development of inhibitors targeting the TpoR-CALR mutant C-terminus interaction. Based on our HDx-MS and functional results, we further propose a complete model of the tetrameric CALR mutant-TpoR complex for both type 1 (CALR del52) and type 2 (CALR ins5) frameshift mutants and highlight key interacting residues. We posit that the interaction between immature N-glycans of TpoR and CALR N-domain occurs concomitantly to binding of negatively charged patches of TpoR D1 domain to CALR mutant C-terminus, the latter providing specificity and additional stability for the interaction. Previous studies suggested that homo-multimerization of CALR mutant proteins preceded binding to TpoR^26^ in the ER and Golgi apparatus^8,34^. This is compatible with our structural models of CALR del52 and CALR ins5 where different faces of the α-helical C-terminus are involved in CALR mutant homodimerization and TpoR interaction. Interestingly, the 19 amino acid insertion in ins5 which increases the distance between the two sites by more than 25Ä does not seem to have a major effect on the complex formation. In conclusion, the work presented herein identifies how frameshift mutations in a master chaperone leads to its conformational remodeling resulting in oncogenic properties. It characterizes specific interactions between CALR frameshift mutants and the TpoR ECD, providing mechanistic understanding of the specificity of this interaction and paving the way for inhibitory therapeutic avenues.

## Methods

### Production and purification of recombinant proteins

Recombinant proteins for human TpoR (hTpoR) D1–D4 and CALR del52-hTpoR D1D2 complex were produced as described previously in Schneider S2 cells^8^ (see also Supplementary Methods). The amino acid sequence of hTpoR D1–D4 starts at Q26 and ends at T489 and this of hTpoR D1D2 starts at Q26 and ends at Q290. Both contains a histidine tag at the C-terminus. The amino acid sequence of hCALR del52 starts at E18 and ends at A411.

Recombinant human CALR wild-type, CALR del52 and its derivatives contain a N-terminal His tag sequence (MGSHHHHHHGSSG) that replaces the CALR signal peptide sequence (aa1–17). In addition, the cysteine 163 was mutated to serine. CALR proteins were produced in *Escherichia coli* (see also Supplementary Methods). The purity and folding of each protein were verified by SDS-PAGE, thermal stability, and chromatography (Supplementary Figs. 1b, c, 2a–g, 6a, b, 8a, b).

### Hydrogen-deuterium exchange mass spectrometry (HDx-MS)

Hydrogen-deuterium exchange mass spectrometry was performed with a Waters nanoAcquity UPLC with HDx technology coupled with Synapt G2-Si. All purified recombinant proteins were used at 20 μM concentration in equilibration buffer (5 mM K_2_HPO_4_, 5 mM KH_2_PO_4_ dissolved in H_2_O, pH 7). For interaction analysis between recombinant mature TpoR D1-D4 and CALR del52, proteins were first mixed together at a 1:1 molarity for 30 min at room temperature followed by 3 h at 4 °C. Proteins were then kept at 0 °C. Labeling was performed with a 20-fold dilution of samples in labeling buffer (5 mM K_2_HPO_4_, 5 mM KH_2_PO_4_ dissolved in D_2_O, pD 7) for 6 different incubation times (0, 0.25, 1, 5, 20 and 60 min) at 20 °C in a randomized order. Final D_2_O concentration was 95% during labeling reaction (3 µL proteins/57 µL labeling buffer). After incubation, the reaction was quenched using a 1:1 dilution in the quench buffer (0.05 M K_2_HPO_4_, 0.05 M KH_2_PO_4_ with 30 mM TCEP, pH 2.3) prior to injection into a pepsin column (Enzymate BEH Pepsin 2.1 × 30 column, Waters CAT. 186007233) with dynamic flowrate of 150–75 µL/min. All mixes were performed automatically by a PAL-RTC robot station. Peptides resulting from the pepsin digestion were captured on a ACQUITY BEH C18 1.7 μM VANGUARD Pre-column (Waters Cat. 186009375), separated on a ACQUITY UPLC C18 1.7 μM 1.0 × 100 mm column (Waters Cat. 186002346) and electrosprayed into the Waters SYNAPT G2-Si quadrupole time-of-flight mass spectrometer. Measurements were performed in HDMSe mode with ion mobility. Lock mass correction was performed with infusion of leucine-enkephalin (*m*/*z* = 556.277). The peptides were identified from triplicates using the PLGS3.0 software (Waters) with a database containing the sequence of all proteins present in the sample and the pepsin used for digestion. The peptides identified were further analyzed with DynamX 3.0 (Waters) using a tolerance of 10 ppm, a maximum length of 35 amino acids, a minimum products per amino acid of 0.2 and requiring that each peptide was identified in 3 out of 3 replicates. All peptides were visually validated based on retention time, drift time and isotopic *m*/*z*. Data was statistically analyzed using Deuteros 2.0 with peptide-level significance testing, which controls for the false discovery rate^35^. A summary of the HDx-MS experimental set-up and raw data are provided in the source data file following reporting suggestions described by Masson and colleagues^36^.

For the representation of HDx-MS data, structures of CALR WT, CALR del52 or CALR ΔC-tail were generated with AlphaFold 2.0^18^. The structures were colored with a gradient from red (less protected, negative value) to blue (more protected, positive values) corresponding to difference in relative fractional uptake (ΔRFU) with dark red and dark blue corresponding to highest H-D differential. The ΔRFU per residue was inserted as b-factor in Pymol 2.4.2. and the structures were colored according to b-factor values with the scale indicated in figure legends. The RFU for a peptide 1 is computed as:

1 $${{{{{{\rm{RFU}}}}}}}_{{{{{{\rm{peptide}}}}}}\,1}=\frac{{{{{{\rm{Uptake}}}}}}{({{{{{\rm{Da}}}}}})}_{{{{{{\rm{peptide}}}}}}\,1}}{{{{{{\rm{Total}}}}}}\,{{{{{\rm{number}}}}}}\,{{{{{\rm{of}}}}}}\,{{{{{\rm{backbone}}}}}}\,{{{{{\rm{amide}}}}}}\,{{{{{{\rm{hydrogen}}}}}}}_{{{{{{\rm{peptide}}}}}}\,1}}$$

The ΔRFU for peptide 1 between condition A and B in then computed as:

2 $${\Delta {{{{{\rm{RFU}}}}}}}_{{{{{{\rm{peptide}}}}}}\,1[{{{{{\rm{A-B}}}}}}]}={{{{{{\rm{RFU}}}}}}}_{1[{{{{{\rm{A}}}}}}]}-{{{{{{\rm{RFU}}}}}}}_{1[{{{{{\rm{B}}}}}}]}$$

### Fourier transformed infrared (FTIR) spectroscopy

0.5 μL of sample was loaded on the diamond crystal of the ATR device of the FTIR spectrometer and quickly dried with a constant, gentle nitrogen flow: elimination of the water molecules prevents overlapping of the large water absorption peaks with the sample’s absorption spectrum. After each spectrum, the crystal was cleaned with water. A background was recorded with a clean crystal before the start of the measurement and before every new sample. FTIR spectra were recorded between 4000 and 600 cm^−1^ at a resolution of 2 cm^−1^. Each spectrum was obtained by taking an average of 128 scans. The FTIR measurements were carried out at room temperature (~22 °C). For each sample, at least four spectra were recorded. All the spectra were preprocessed as follows. The water vapor contribution was subtracted with 1956–1935 cm^−1^ as reference peak. All spectra were then baseline-corrected and normalized as follows. Straight lines were interpolated between the following frequencies: 3700 3000 2800 1720 1480 1204 980 cm^−1^. Then, they were subtracted from the spectrum. Normalization for equal area was applied between 1720 and 1480 cm^−1^. Using a database of 50 protein containing as little fold redundancy as possible, an ascending stepwise method was applied to determine the protein secondary structure. It was demonstrated that three wavenumbers contain all the nonredundant information related to the secondary structure content. The standard error of prediction in cross-validation obtained using the 50 protein database was 5.7% for the α-helix and 6.7% for the β-sheet, 3.2% for turns and 8% for random^37,38^. Detailed protocol and unprocessed data are provided in Supplementary Methods and Supplementary Fig. 4a, respectively.

### Molecular dynamics and docking simulations

Sequences of TpoR extracellular and transmembrane regions and of CALR Del52 were profiled for secondary structure, intrinsic disorder and accessibility propensities with state-of-the-art predictors^39–46^. Closest templates were retrieved with Phyre 2^47^. Modeller 9.21^48^, AlphaFold2 and Rosetta Folding^18^. For the study of interactions between CALR mutant tail and TpoR D1D2, docking trials were performed using three main start configurations of the complex based on the acidic areas of TpoR set as inputs in HADDOCK^23,49^ for TpoR D1D2-CALR del52 mutant tail complex optimizations searches. The top configurations were further optimized using 500 ns molecular dynamics runs performed with OpenMM version 7.4.1^50^ using a Monte Carlo Barostat, at 300 K, using a Langevin integrator with 1ps-1 friction coefficient and a 2 fs timestep and the FF14SB Force Field^51^ to obtain 3 final poses in which the last residues of the mutant become unfolded. Free energy was estimated by both a knowledge-based method, using PRODIGY server^24^ and a physical MD estimation approach based on 3 simulations for each pose, using the MM-GBSA method^25^ at 150 mM salt concentration, implemented in AMBER20^52^. Conformational discretization for microstate analysis was performed using Time-Lagged Independent Component Analysis (TICA). The backbone dihedral angles of the CALR del52 mutant C-terminus molecule were used as input coordinates for TICA. TICA and free energy surfaces were computed using the PyEMMA (2.5.11) package^53^, resulting plots were generated using the Matplotlib (3.5.1) package^54^. The inflection core state (InfleCS) clustering method^55^ was used to cluster the two transformed coordinates with the highest eigenvalues and the associated cluster centers were plotted on the corresponding free energy surface. Clustering was performed using 10 components, re-estimation of the same Gaussian mixture model was done 5 times.

Templates from AlphaFold2 and Rosetta Folding were used to effectively build the tetrameric 3D models and identify the interaction interface between the two CALR mutants. HDx-MS data was used to identify contacts between TpoR and CALR del52 in the formation of the tetramer complex. The ER specific G1M9 glycans of TpoR in contact to CALR were modeled with Glycopack^56^ in the configuration consistent with NMR data^57^ while the rest are of complex type, built in agreement with SAGS Database (https://sags.biochim.ro/)^58,59^. The HDx-MS identified contacts and the solid-NMR data on the TM region configuration of TpoR dimer were used as constraints in generating the overall 2CALR-2TpoR model. This glycoproteic tetramer was immersed into a full-atom representation of the environment - consisting in a lipid bilayer of 1907 POPC molecules accommodating the TM region of TpoR and in 478479 TIP3P water molecules, 1328 chloride and 1402 sodium ions describing the solvent region hydrating the rest of the tetramer using the CHARMM-GUI server^60^. This overall system consisting of ~ 1 million atoms was subjected to a mild simulated annealing procedure consisting in a start minimization, heating to 300 K followed by cooling to 0 K and final extended minimization, using NAMD v.2.13^61^ CHARMM36 forcefield^62–64^. The same procedure was used to build TpoR-CALR ins5 complex. The TpoR-CALR-del52/ins5 models were further subjected to 3 molecular dynamics runs to explore the configuration sample space. More detailed protocols, including intermediate modeling steps, free energy estimates and detailed TICA analysis are presented in Supplementary Methods.

### Transcriptional dual luciferase assay

Transcriptional dual luciferase assays were performed as described^5^. Briefly, HEK293T were transiently transfected with empty vector or human TpoR WT with indicated CALR species. In both cases, SpiLuc reporter was used as a readout of STAT5 transcriptional activation and pRLTK was used as an internal control (Promega). Cells were stimulated, or not, with 25 ng/mL of rhTpo (Milteneyi Biotec) as indicated.

### Western blotting and co-immunoprecipitation

HEK293T were plated in 10 cm dishes and transiently transfected with cDNA coding for the indicated constructs. Confluent cells were lysed 48 h post transfection with NP-40 buffer. After pre-clearing, samples were incubated with anti-FLAG antibody (Genscript, Cat. No. A00187) at 2 µg/mL or corresponding isotype control (Genscript Cat. No. A01730) overnight at 4 °C. Bound proteins were pulled down with 40 µL/mL of rProtein G Agarose (ThermoFisher, 20397) for 3 h at 4 °C. Samples were then centrifuged, washed three times and immunoprecipitated proteins were analyzed by SDS-PAGE followed by revelation with an anti-HA antibody (Cell Signaling, C29F4) for HA-hTpoR, HA-CALR del52 or anti-CALR mutant tail (Clone SAT602, MyeloPro GmBH).

### Antibodies

Anti-FLAG tag (Genscript No. A00187) 3 µg/mL whole cell lysate for immunoprecipitation and 1:500 dilution for western blots. Anti-HA tag clone C29F4 (Cell Signalling #3724) 1:1000 dilution for western blots. Mouse IgG control (Genscript No. A01730) 3 µg/mL whole cell lysate for immunoprecipitation. Anti-mutant CALR (Myelopro, Clone SAT602) 1.3 µg/mL for western Blot. Anti-rabbit IgG, HRP-linked (Cell Signaling #7074) 1:5000 for western blots. Anti-mouse IgG, HRP-linked (Cell Signaling #7076) 1:5000 for western blots.

### Nano-bioluminescence energy transfer (BRET)

Nano-bioluminescence resonance energy transfer (BRET) was performed as previously described^8^. The specificity of the interaction was validated following the manufacturer’s instruction (Promega), see Supplementary Methods and Supplementary Fig. 7.

### Microscale thermophoresis (MST)

Recombinant human CALR del52 was labeled with NHS- chemistry according to the manufacture’s instruction (Protein Labeling Kit RED-NHS 2^nd^ Generation, NanoTemper Technology), referred as the “target protein”. Briefly, the target protein (at 10 μM) was incubated with the dye solution in the labeling buffer (130 mM NaHCO_3_, 50 mM NaCl, pH 8.2–8.3) for 1 h on ice. The dye carries a reactive NHS-ester group that reacts with primary amines (lysine residues) to form covalent bonds. The surplus of the dye not bound to the target protein was removed through passage on a resin column prior to elution of the target in the equilibration buffer (Tris-HCL 1 M, pH 7.6). For MST measurement, the CALR del52-NHS was used at 20 nM final concentration in MST buffer (Tris based supplemented with 0.01% of tween 20).

TpoR D1–D4 (the “ligand”) remained label free. Serial dilutions (0.15 nM to 5 μM) of the ligand (TpoR D1-D4) were performed to titer the target protein (CALR del52). The measurements were performed on a NanoTemper monolith NT.115 instrument (NanoTemper technologies, Germany) at 40% LED and medium MST-power with a standard 5 s. before, MST-on for 30 s. and 5 s. after MST-off.

### Proliferation assay

Ba/F3 were transduced with human TpoR in pMX-IRES-GFP and CALR variants or an empty vector (pMSCV-IRES-mCherry) and sorted by FACS for similar levels of GFP and mCherry. 250,000 cells were washed and seeded in 10 mL RPMI, 10% FBS without cytokine and counted each day using a Coulter automated cell counter in triplicates. The experiments were performed in three different biological replicates (*N* = 3).

### Mutagenesis

All mutants were made alternatively using the QuickChange (Agilent), the KLD enzyme mix (NEB) procedure following the manufacturer instruction or purchased from Genscript. All constructs were verified by sequencing.

### Crosslinking

HEK293T were plated in 6 wells plates and co-transfected with indicated constructs. TpoR L508C was truncated after Box 2 to avoid non-specific crosslinking of intracellular cysteines. 48 h post transfection, cells were harvested without trypsinization and washed in PBS. Cells were then re-suspended and incubated for 15 min at room temperature in crosslinking buffer (PBS 1 mM MgCl_2_, 0.1 mM CaCl_2_) with 100 μM N-ethylmaleimide (NEM, ThermoFisher Cat.:23030) to avoid non-specific crosslinking of extracellular cysteines. 200 ng/mL of rhTpo was added in the indicated condition. Samples were mixed gently and further incubated for 15 min at room temperature. Cells were then centrifuged for 5 min at 500×*g* and re-suspended in crosslinking buffer with 100 μM o-phenylene dimaleimide (o-PDM, Sigmaaldrich) for 10 min at room temperature. Cells were further centrifuged 5 min at 500×*g* and re-suspended in lysis buffer (NP-40, 2% ß-mercaptoethanol) with protease inhibitor cocktail. Cell lysates were analyzed by SDS-PAGE in denaturing and reducing conditions with anti-HA antibody.

### Cell lines and cell culture

HEK293T were obtained from the American Type Culture Collection (ATCC) (CRL-3216™). They were cultivated in Dulbecco’s Modified Eagle Medium (Gibco) supplemented with 10% fetal bovine serum (Gibco). Ba/F3 cell lines were obtained previously after isolation of clones with pro-B lymphocyte characteristics^65^ and were transferred from laboratory of Prof. Harvey Lodish (Whitehead Institute, MIT) to Ludwig Institute for Cancer Research, Brussels Branch. Ba/F3 were cultured in Roswell Park Memorial Institute (RPMI) medium (Gibco) supplemented with 10% FBS (Gibco) and 0.5 ng/mL of murine IL-3 (RnDsystems). IL-3 was removed by washing the cells at three times with PBS prior to experiments.

### Reporting summary

Further information on research design is available in the Nature Portfolio Reporting Summary linked to this article.

## Supplementary information

Supplementary Information

Description of Additional Supplementary Files

Supplementary Data 1

Supplementary Data 2

Reporting Summary

## Data Availability

Mass spectrometry data was deposited on the ProteomeXchange repository (https://www.proteomexchange.org) under accession number PXD034131. PDB files of the molecular dynamics simulations were deposited on Figshare (https://figshare.com/s/b4ceb87fdce1f242e469 and https://figshare.com/s/9033970b5a1d3f8d6fa7). The detailed protocols to produce the recombinant proteins used in this study are provided as Supplementary Methods. We can provide plasmids coding for each protein upon request after signing of a material transfer agreement and payment of a fee corresponding to shipment and preparation costs. Source data are provided with this paper. The source data file provides the raw data and reporting of HDx-MS experiments following the guidelines suggested by Masson et al.^36^ in addition to source data for all other experiments. Source data are provided with this paper.

## Source data

Source Data
