# Supplementary material for: Oncogenic CALR mutant C-terminus mediates dual binding to the thrombopoietin receptor triggering complex dimerization and activation
Source: Nat Commun. 2023 Apr 5;14:1881. doi: 10.1038/s41467-023-37277-3 (PMC10076285; doi:10.1038/s41467-023-37277-3)
Supplement: Supplementary file 1 — Supplementary Information [file 41467_2023_37277_MOESM1_ESM.pdf]

Supplementary Information

Oncogenic CALR Mutant C-terminus Mediates Dual Binding to the Thrombopoietin Receptor Triggering Complex Dimerization and Activation

Nicolas Papadopoulos[1,2], Audrey Nédélec[1,2], Allison Derenne[3], Teodor Asvadur Şulea[4], Christian Pecquet[1,2], Ilyas Chachoua[1,2,5], Gaëlle Vertenoeil[1,2], Thomas Tilmant[6], Andrei-Jose Petrescu[4], Gabriel Mazzucchelli[6], Bogdan I. Iorga[7], Didier Vertommen[2,8], Stefan N. Constantinescu[1,2,9,10]*

*Corresponding Author: Stefan N. Constantinescu

Email: Stefan.constantinescu@bru.licr.org

[1]Ludwig Institute for Cancer Research Brussels, Brussels, Belgium.

[2]Université Catholique de Louvain and de Duve Institute, Brussels, Belgium.

[3] Spectralys Biotech SRL, rue Auguste Piccard 48, 6041 Gosselies, Belgium

[4]Department of Bioinformatics and Structural Biochemistry, Institute of Biochemistry of the Romanian Academy, Splaiul Independentei 296, Bucharest 060031, Romania.

[5]Bilkent University, Department of Molecular Biology and Genetics, Ankara, Turkey

[6]Mass Spectrometry Laboratory, MolSys Research Unit, Universiy of Liège, 4000, Liège, Belgium.

[7]Université Paris-Saclay, CNRS, Institut de Chimie des Substances Naturelles, UPR 2301, Gif-sur-Yvette, France

[8] de Duve Institute and MASSPROT platform, Brussels, Belgium

[9]Walloon Excelence in Life Sciences and Biotechnology, WELBIO, avenue Pasteur, 6, 1300 Wavre, Belgium.

[10]Ludwig Institute for Cancer Research, Nuffield Department of Medicine, Oxford University, Oxford, UK.

Supplementary Figures

a

CALR species	Theoretical MW	Measured Tm (°C)
CALR WT	47.8 kDa	49.7
CALR del52	47.3 kDa	45.7
CALR del52 A394*	45.3 kDa	40.3
CALR del52 ΔC-tail	41.7 kDa	48.8
CALR del52 Y109F/D135L	47. 3 kDa	42.5

b

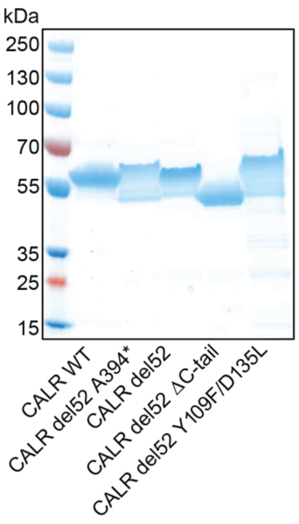

c

d

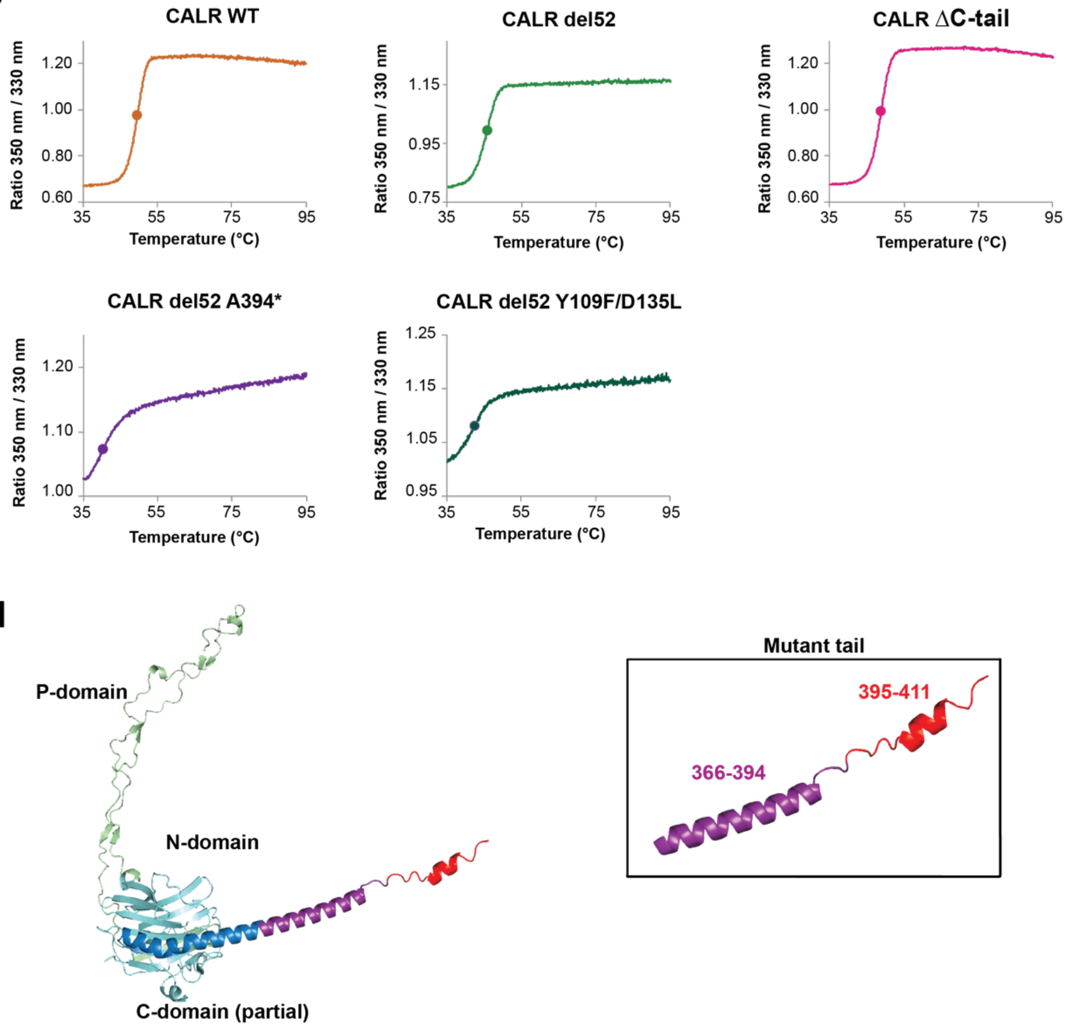

Supplementary Figure 1. CALR species used in this study.

a. Summary of recombinant CALR proteins used in HDx-MS and FTIR experiments. Theoretical molecular weight (MW) and T_m measured with Tycho NT.6 instrument (NanoTemper). **b**. Coomassie Blue staining of CALR WT, CALR del52 and indicated variants. Purified samples were analyzed by SDS-PAGE in denaturing and reducing conditions and stained with Coomassie Blue for total protein detection**.** Representative gel from 3 experiments. **c.** Thermal stability of purified proteins. The graphs represent the 350/330 nm intrinsic fluorescence from Trp and Tyr residues at different temperatures. The S-shaped curve is typical of well-folded proteins as the accessibility of Tyr and Trp residues gradually increases upon temperature-induced protein unfolding. The T_m is computed as the temperature at which half of the proteins in the sample are denatured. Source data are provided as a Source data file. **d.** Structure prediction using AlphaFold 2.0[1] of CALR del52 mutant protein. The mutant C-terminus is highlighted. The N-domain is shown in light blue, the P-domain in green, the partial C-domain of CALR WT in dark blue and the mutant tail in magenta until position 394 and in red from 395 to 411.

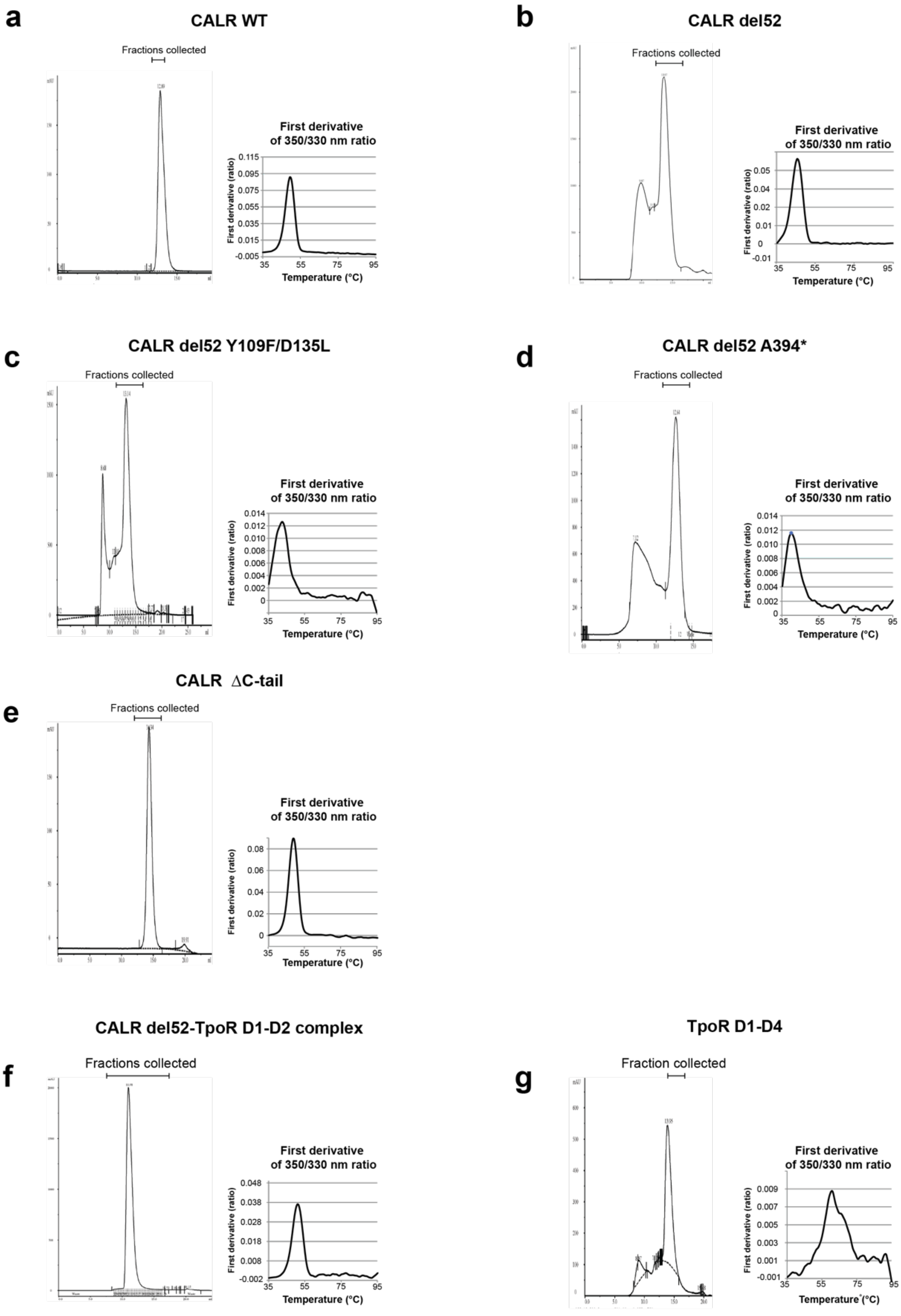

Supplementary Figure 2. Size-exclusion chromatography and purity of recombinant proteins.

a-g. Left: Size-exclusion chromatograph of indicated proteins in HDx equilibration buffer. The final fraction collected for is indicated. Right: Profile of the first derivative of the 350/330 nm ratio of indicated proteins. The position of the peak on the X axis indicates the Tm of each protein. The shape of the first derivative ratio is used as an additional indicator of protein purity.

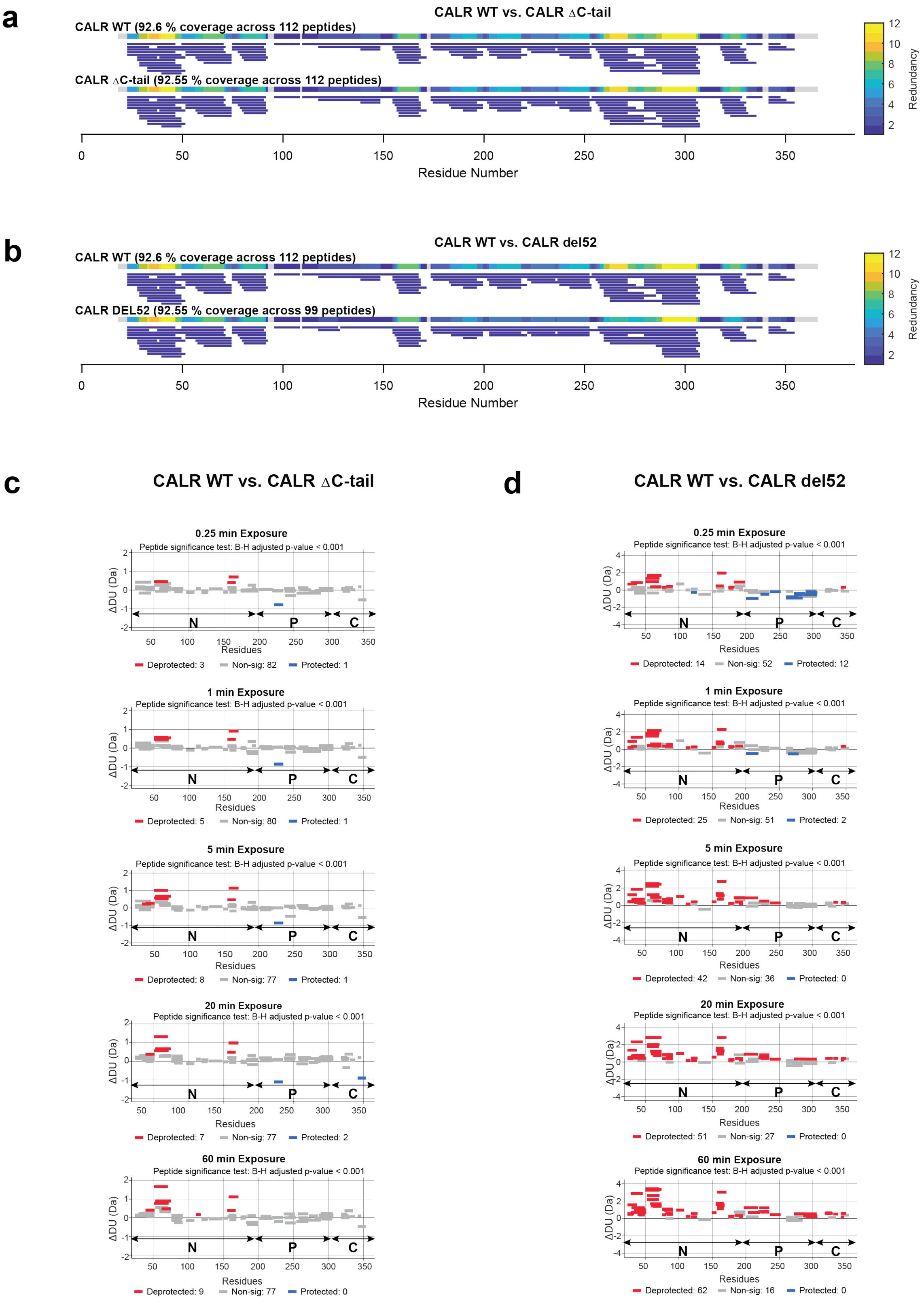

Supplementary Figure 3. HDx-MS footprints of CALR WT, CALR del52 and CALR ΔC-tail

a-b. Sequence coverage obtained for the H-D exchange analysis between CALR WT and CALR ΔC-tail (a) and between CALR WT and CALR del52 (b). The percentage of sequence

coverage and the number of peptides detected are indicated. **c-d.** Wood's plots linked to Figure 1C-D generated with Deuteros 2.0[2]. Each bar (wood) represents the H-D exchange differential for a single peptide between CALR WT and CALRΔC-tail (c) or between CALR WT and CALR del52 (d) at 0,0.25,1,5,15 or 60 minutes incubation in deuterium. Peptides in red (deprotected) or blue (protected) have significant differential H-D exchange ($p<0.001$) with the peptide-level significance testing ($n = 3$) as described [2]. The N-, P- and C-domains of CALR are indicated on the plots by letters N, P and C, respectively. Source data are provided as a Source data file.

a

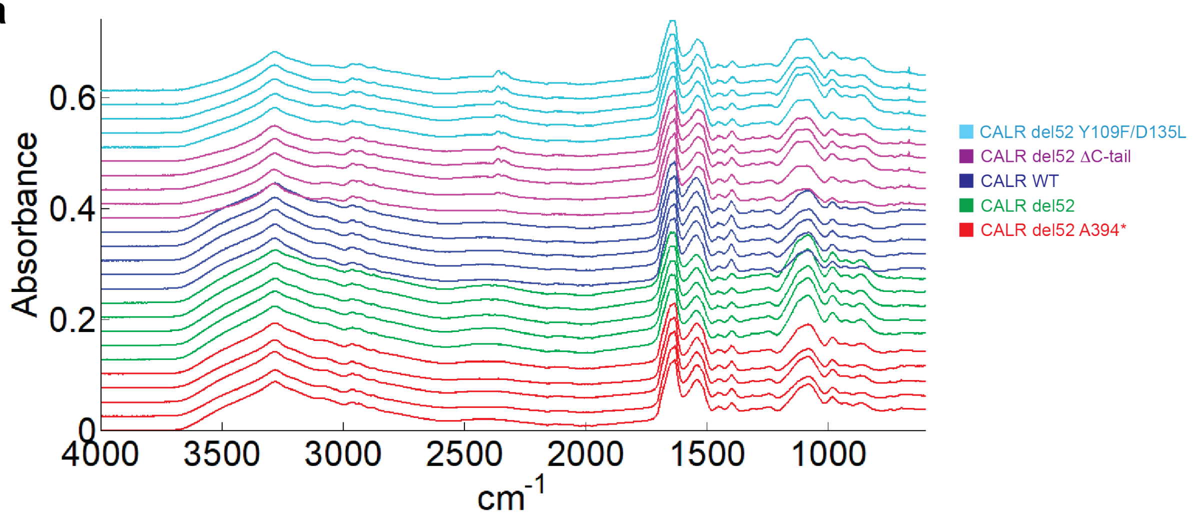

b

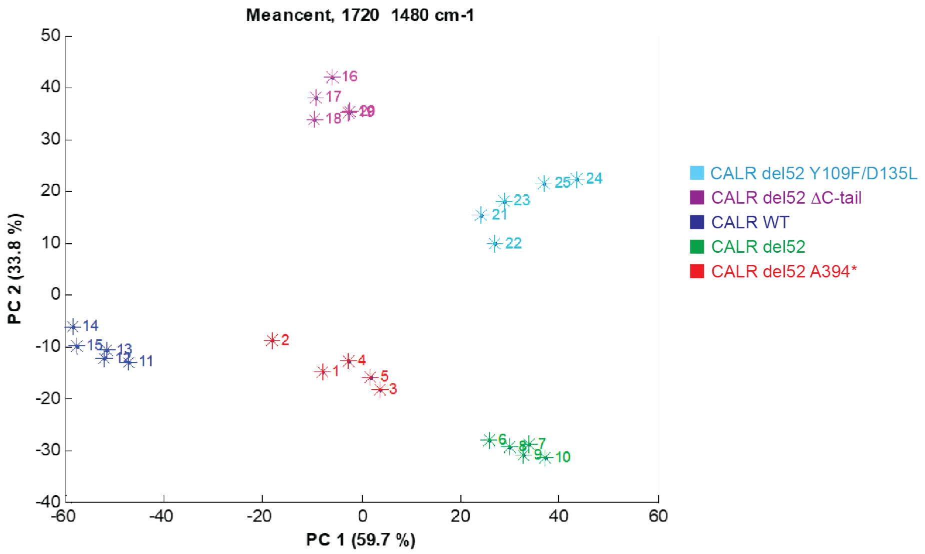

c

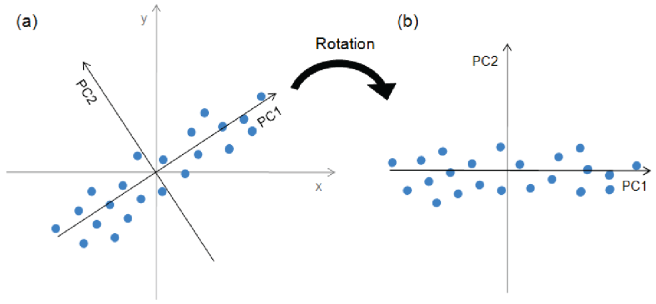

Supplementary Figure 4. FTIR spectra of CALR species.

a. Raw data - all FTIR spectra recorded (without any preprocessing step) to analyse the structure. Each sample is identified by a unique color indicated in the legend. For better readability, spectra were offset along the absorbance axis. **b.** PCA score plot depicting the projection of the 25 individual preprocessed FTIR spectra of the WT and mutant proteins in

the PC1-PC2 space performed on the 1720-1480 cm^{-1} spectral region. Each star stands for one spectrum. For the sake of clarity, a colour is associated with each sample but the analysis is completely unsupervised. Percentages on the axis labels indicate the variance described by PC1 (59.7%) and PC2 (33.8%). A mean centering (subtraction of the arithmetic mean from all the spectra) was applied on this set of data. **c.** (a) Representation of the original data in terms of the two axes x and y. Each point represents one IR spectra. (b) As a result of the PCA the axes are rotated and the data will be represented in the two-dimensional principal component space. Thereby PC1 represents the largest variance in the data.

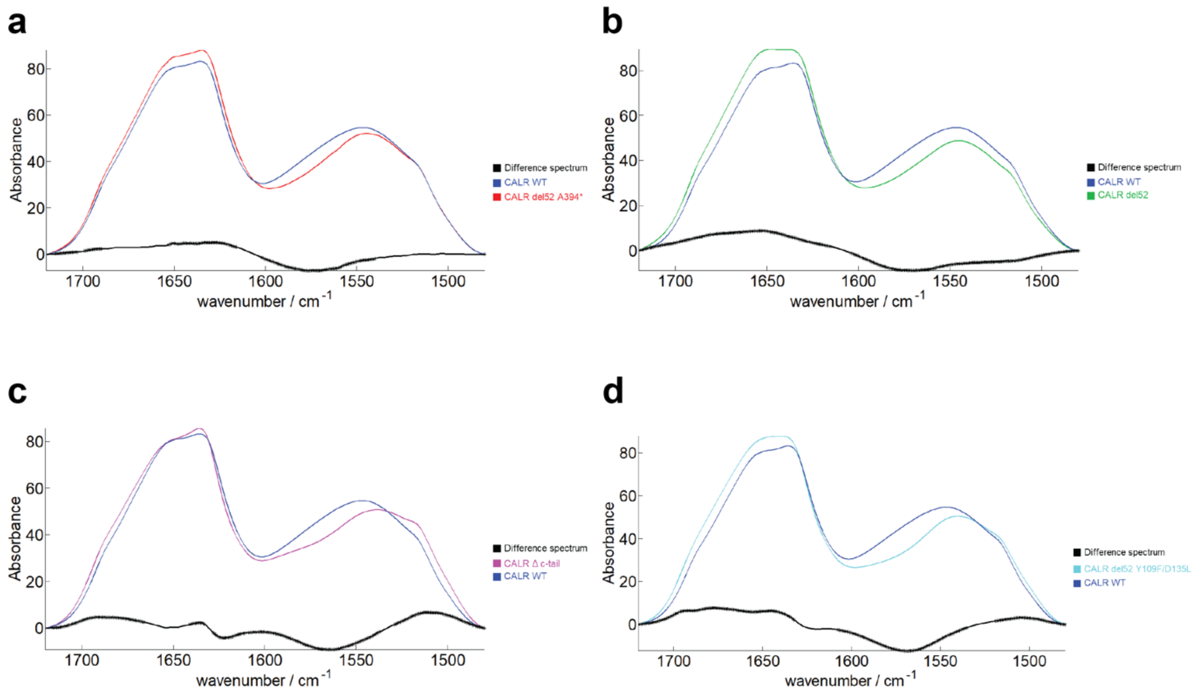

Supplementary Figure 5. Raw FTIR spectra of CALR species.

a-d. Comparison of the processed mean spectra recorded. The difference between the two mean spectra indicated is shown in black – zoom on the spectral region related to proteins absorption (1720-1480 cm^{-1}). A Student's t-test was carried out at each wavenumber with a confidence level α=0.1%. The significant spectral differences are revealed with black stars on the difference spectrum. Each spectrum is identified by a unique colour indicated in the legend.

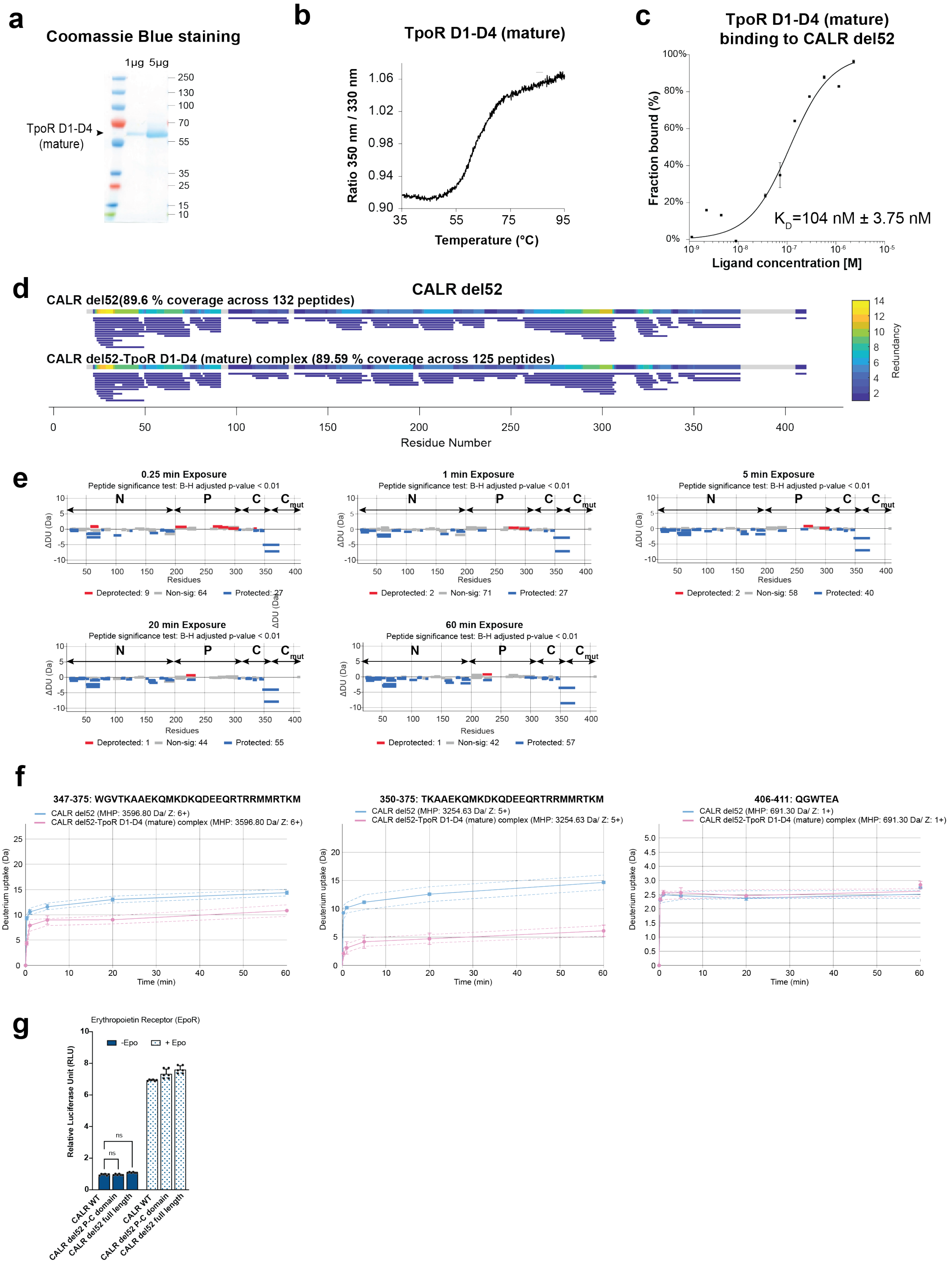

Supplementary Figure 6. CALR mutant C-terminus specifically interacts with TpoR in absence of immature N-glycans.

a. Coomassie Blue staining of TpoR D1-D4 with mature N-glycans. Purified TpoR D1-D4 was analyzed by SDS-PAGE in denaturing and reducing conditions and stained with Coomassie

Blue for total protein detection. Representative gel from 3 experiments. **b.** Thermal stability of TpoR D1-D4 with mature N-glycans. The graphs represent the 350/330 nm intrinsic fluorescence from Trp and Tyr residues at different temperatures. The S-shaped curve is typical of well-folded proteins as the accessibility of Tyr and Trp residues gradually increases upon temperature-induced protein unfolding. Source data are provided as a Source data file. **c**. Microscale Thermophoresis between CALR del52 and TpoR D1-D4 with mature N-glycans. CALRdel52 was labeled with RED-NHS 2nd Generation chemistry according to the manufacture's instruction (NanoTemper Technology). The curve corresponds to the mean (± SD) of two independent experiments following the fluorescence of the target protein (CALRdel52-NHS) with titration of the the ligand (TpoR D1-D4). While the concentration of the target is kept constant at 20 nM, the ligand concentration ranges from 5uM and 0,15 nM. The binding curve represents the percentage of bound fraction of CALR del52 to TpoR D1-D4 and yields a K_D of 104 nM ± 3.75 nM. Source data are provided as a Source data file. **d.** Sequence coverage obtained for the H-D exchange analysis between CALR del52 alone and CALR del52 in complex with TpoR D1-D4 with mature N-glycan. The percentage of sequence coverage and the number of peptides detected are indicated. **e.** Wood's plots linked to Figure 2A generated with Deuteros 2.0[2]. Each bar (wood) represents the H-D exchange differential for a single peptide between CALR del52 alone and CALR del52 in complex with TpoR D1-D4 with mature N-glycans at 0,0.25,1,5,15 or 60 minutes incubation in deuterium. Peptides in red (deprotected) or blue (protected) have significant differential H-D exchange ($p<0.001$) with the peptide-level significance testing (n = 3) as described [2]. The N-, P- and WT C and mutant C-domains of CALR are indicated on the plots by letters N, P and C and C_{mut}, respectively. Source data are provided as a Source data file. **f**. Deuterium uptake (Da) of the indicated peptides of the mutant C-terminus from CALR del52 alone or in complex with TpoR D1D4 (with mature N-glycans) at 5 different exchange time points. The dotted lines represent standard deviation (SD), the full line represents average of triplicates. Source data are provided as a Source data file. **g.** STAT5 transcriptional activity in presence of EpoR and indicated CALR truncations. HEK293T were transiently transfected with human EpoR and CALR truncations along with cDNAs coding for STAT5, JAK2 and SpiLuc Firefly luciferase reporter reflecting STAT5 transcriptional activity and normalized with a control reporter (pRLTK) containing Renilla luciferase. Data represent mean $\pm$ SD (n = 6 biologically independent samples over 2 independent experiments). Data were analyzed with two-ways ANOVA followed by Sidak multiple comparison test. ns: non-significant ($p>0.05$). Source data are provided as a Source data file.

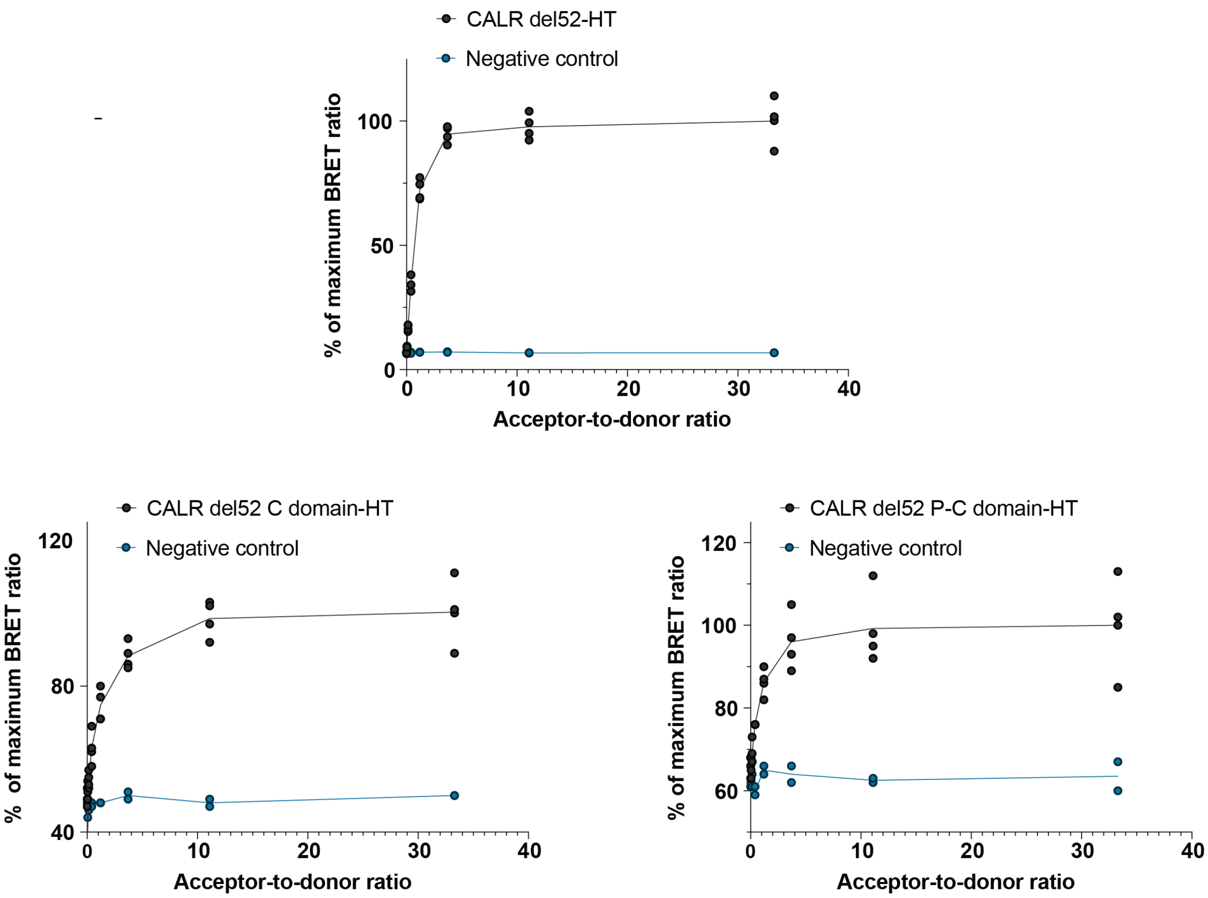

Supplementary Figure 7. Donor Saturation Assay between TpoR-NanoLuc and CALR del52-HaloTag.

Donor Saturation Assay between TpoR-NanoLuc and indicated CALR del52-HaloTag (HT) construct. HEK293T were co-transfected with fixed amount of donor (TpoR-NanoLuc) and increasing ratios of acceptor (HaloTag fusion proteins). The negative control corresponds to a HaloTag protein non-fused to TpoR. A specific BRET signal will increase in a hyperbolic manner before reaching a plateau. A non-specific interaction will be less intense and increase linearly without reaching a plateau. The shape of the curve in a Donor Saturation Assay provides a control for the specificity of the interaction according to the manufacturer instruction (Promega).

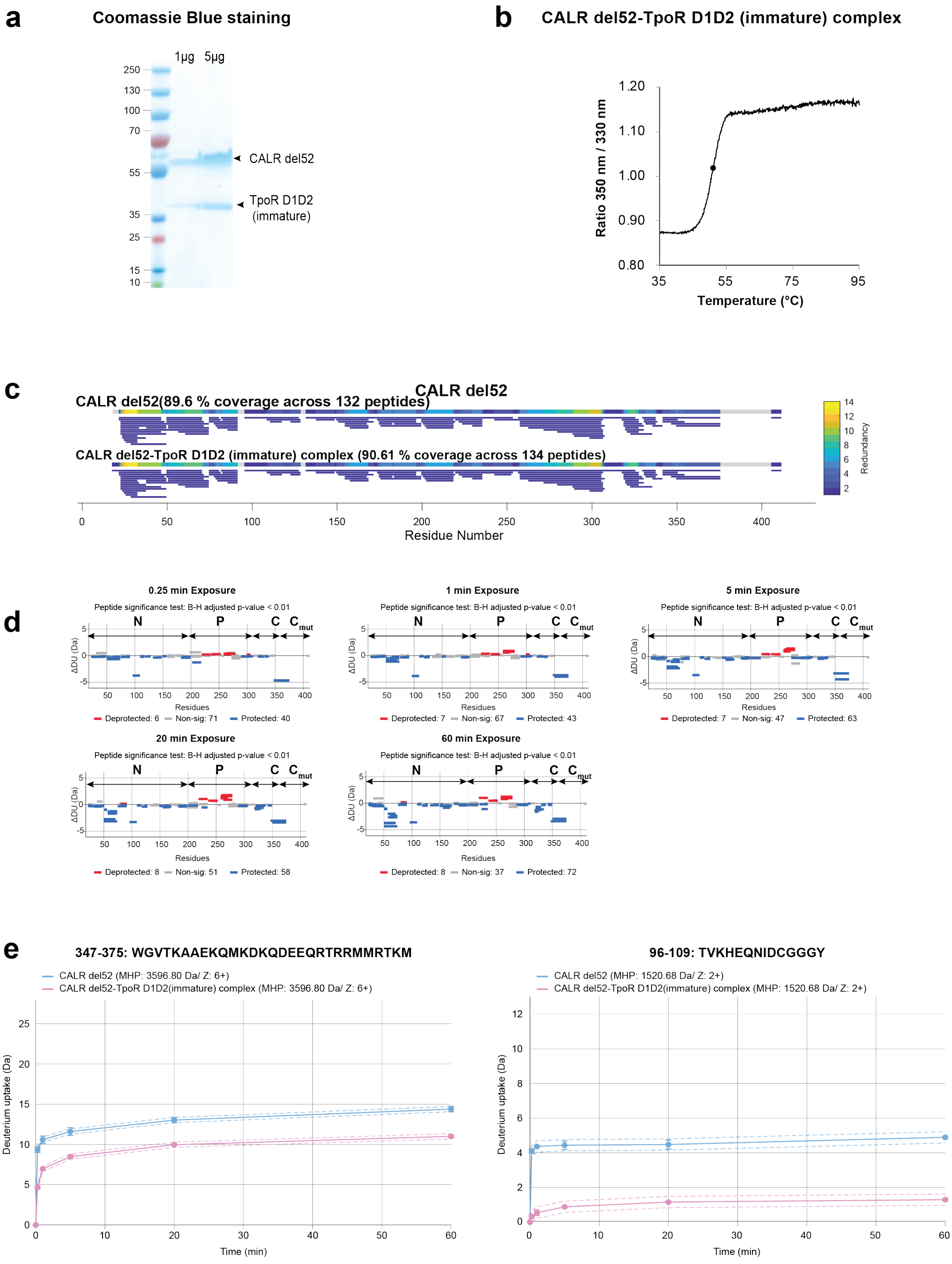

Supplementary Figure 8. Interaction between CALR mutant and TpoR in presence of immature N-glycans.

a. Coomassie Blue staining of the complex CALR del52-TpoR D1D2 with immature N-glycans on Asn117. The purified complex was analyzed by SDS-PAGE in denaturing and reducing

conditions and stained with Coomassie Blue for total protein detection. Representative gel from 3 experiments. **b.** Thermal stability of the CALR del52-TpoR D1D2 complex. The graphs represent the 350/330 nm intrinsic fluorescence from Trp and Tyr residues at different temperatures. The S-shaped curve is typical of well-folded proteins as the accessibility of Tyr and Trp residues gradually increases upon temperature-induced protein unfolding. Source data are provided as a Source data file. **c.** Sequence coverage obtained for the H-D exchange analysis between CALR del52 alone and the CALR del52-TpoR D1D2 complex with immature N-glycans on TpoR Asn117. The percentage of sequence coverage and the number of peptides detected are indicated. **d.** Wood's plots linked to Figure 3E generated with Deuteros 2.0[2]. Each bar (wood) represents the H-D exchange differential for a single peptide between CALR del52 alone and the CALR del52-TpoR D1D2 complex at 0,0.25,1,5,15 or 60 minutes incubation in deuterium. Peptides in red (deprotected) or blue (protected) have significant differential H-D exchange ($p<0.001$) with the peptide-level significance testing ($n = 3$) as described [2]. The N-, P- WT C and mutant C-domains of CALR are indicated on the plots by letters N, P and C and C_{mut}, respectively. Source data are provided as a Source data file. **e**. Deuterium uptake (Da) of the indicated peptides of the mutant C-terminus (left) or N-glycans binding sites (right) from CALR del52 alone or in complex with TpoR D1D2 (with immature N-glycans on Asn117) at 5 different exchange time points. The dotted lines represent standard deviation (SD), the full line represents average of triplicates. Source data are provided as a Source data file.

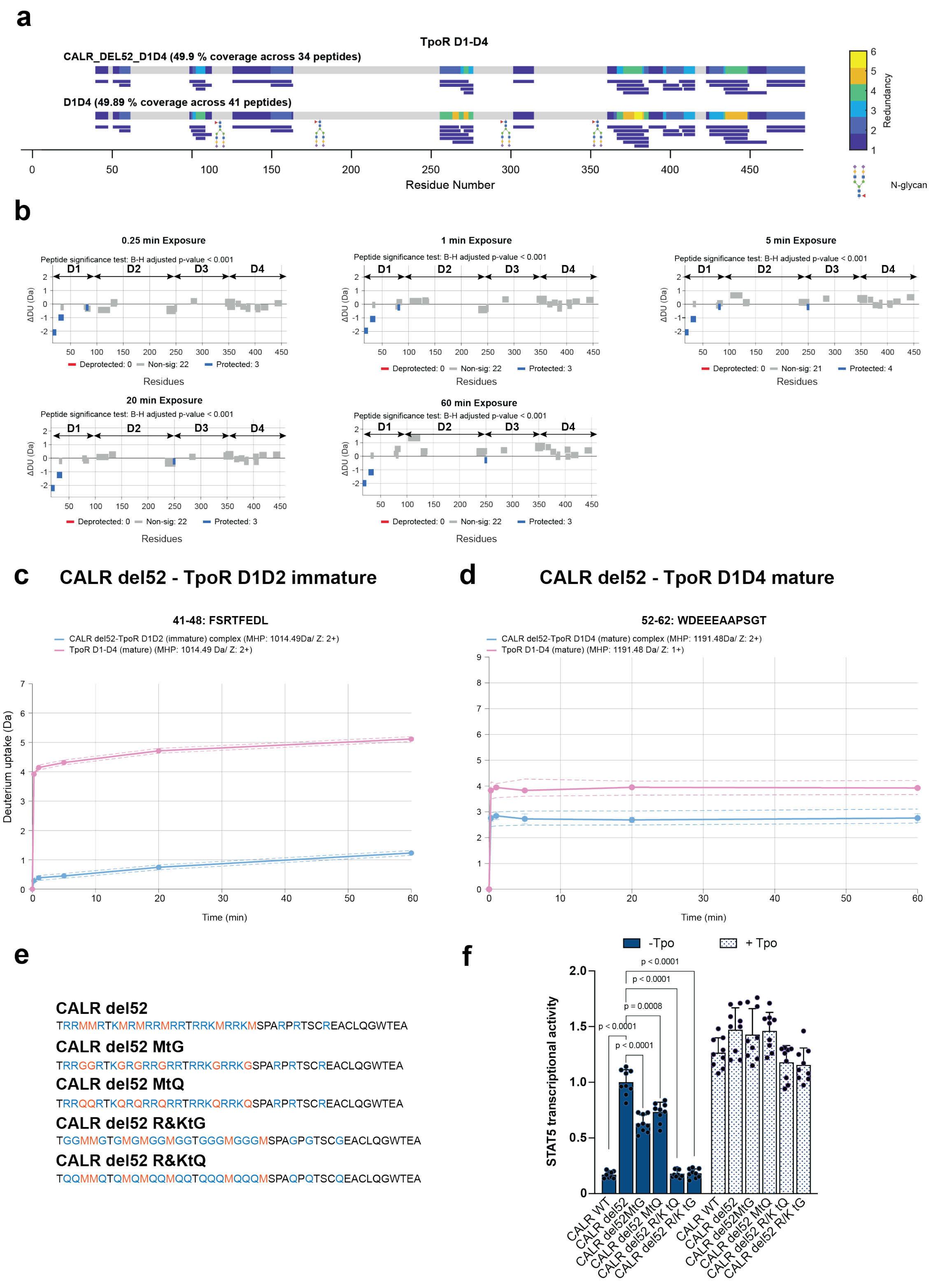

Supplementary Figure 9. Interaction between CALR mutant C-terminus and acidic patches on TpoR D1 domain.

a. Sequence coverage obtained for the H-D exchange analysis between TpoR D1-D4 alone (with mature N-glycans) and in presence of CALR del52. The percentage of sequence coverage and the number of peptides detected are indicated. The peptide signal sequence is

not shown. The sites of N-glycans attachment are indicated. **b.** Wood's plots generated with Deuteros 2.0[2]. Each bar (wood) represents the H-D exchange differential for a single peptide between TpoR D1-D4 alone and the CALR del52-TpoR D1-D4 complex at 0,0.25,1,5,15 or 60 minutes incubation in deuterium. Peptides in red (deprotected) or blue (protected) have significant differential H-D exchange ($p<0.001$) with the peptide-level significance testing as described [2]. The D1, D2,D3 and D4 domains of TpoR are indicated. Source data are provided as a Source data file. **c-d**. Deuterium uptake (Da) of (C) the FSRTFEDL peptide from TpoR D1-D4 alone and from the CALR del52-TpoR D1D2 complex and (D) the WDEEEAAPSGT peptide from TpoR D1-D4 alone and in presence of CALR del52 at 5 different exchange time points. The dotted lines represent standard deviation (SD), the full line represents average of triplicates. Source data are provided as a Source data file. **e.** Sequence of CALR del52 C-terminus and mutants as indicated. **f.** STAT5 transcriptional activity with human TpoR and indicated CALR del52 mutants. HEK293T were transiently transfected with human TpoR and CALR mutants along with cDNAs coding for STAT5, JAK2 and SpiLuc Firefly luciferase reporter reflecting STAT5 transcriptional activity and normalized with a control reporter (pRLTK) containing Renilla luciferase. Data represent mean $\pm$ SD ($n = 9$ biologically independent samples over 3 independent experiments). Data were analyzed with two-ways ANOVA followed by Sidak multiple comparison test. Source data are provided as a Source data file.

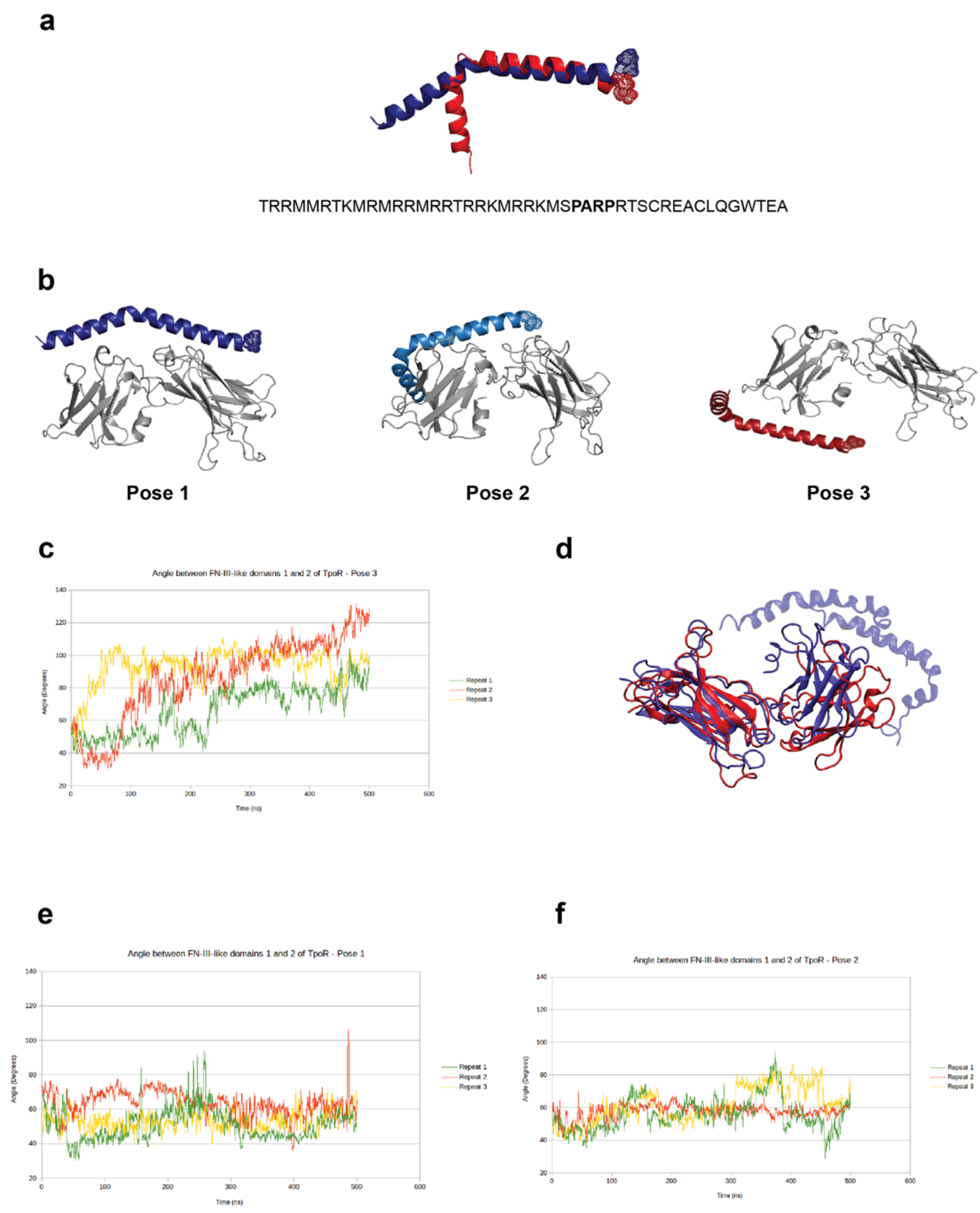

Supplementary Figure 10. Molecular Dynamics between CALR mutant C-terminus and TpoR D1D2 domains.

a. Representative conformations of the main 2 clusters of CALR del52 mutant C-terminus with the bending region in bold in sequence. **b.** Poses generated by HADDOCK. The best docking complex was chosen, as ranked by the HADDOCK score. Render done in PyMOL[40] v. 2.2.3 **c.** Timeseries of the angle between the two FN-III-like domains of the membrane distal TpoR domain, moving average with 0.5 ns window. The C_α atoms of Q26, S129 and D283 were used to compute the angle. **d.** Conformation superposition of the TpoR-CALR del52 mutant C-terminus structure before (purple/blue) and after (red) 500 ns of molecular dynamics. **e-f.**

Timeseries of the angle between the two FN-III-like domains of the membrane distal TpoR domain for pose 1 and 2, moving average with 0.5 ns window. The C_α atoms of Q26, S129 and D283 were used to compute the angle.

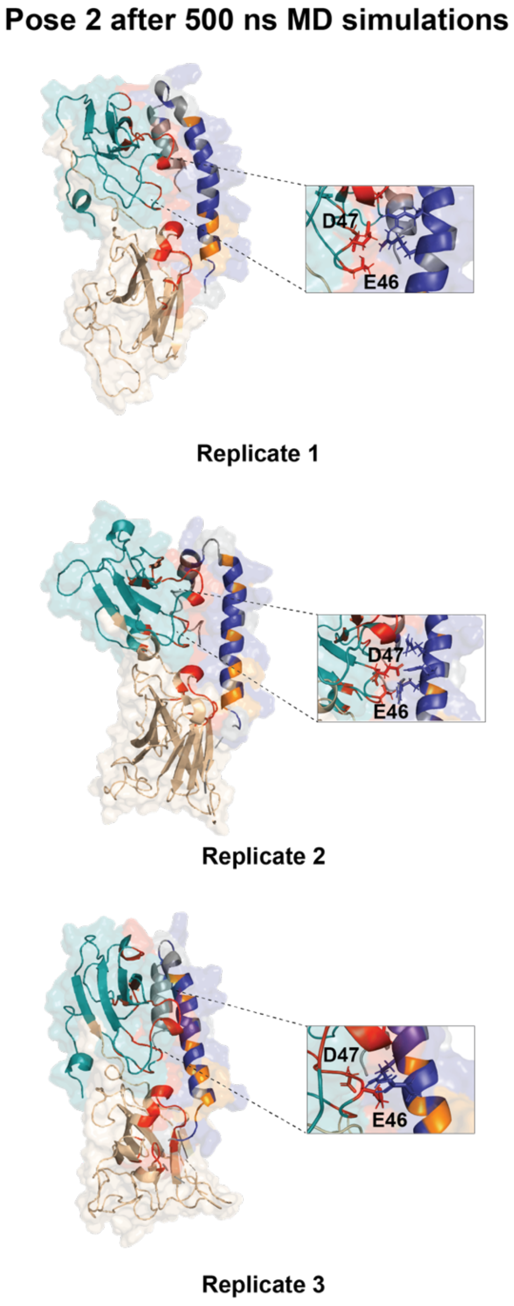

Supplementary Figure 11. Final frames Molecular Dynamic simulations between CALR mutant C-terminus and TpoR D1D2 domain.

Last frame of triplicates unconstrained molecular dynamics simulations (500 ns) for pose 2. The last frame is shown for each replicate independently. The D1 and D2 domain of TpoR are shown in blue/deepteal and beige, respectively. The Arg and Lys of CALR del52 C-terminus are shown in dark blue, the Met in orange and other residues in grey. Residues of TpoR that with a distance < 3.5 Ä from CALR del52 C-terminus are shown in red. The E and F of the

^{44}TFED^{47} motif that interact with Arg of CALR del52 C-terminus are highlighted. Render done with Pymol 2.4.2.

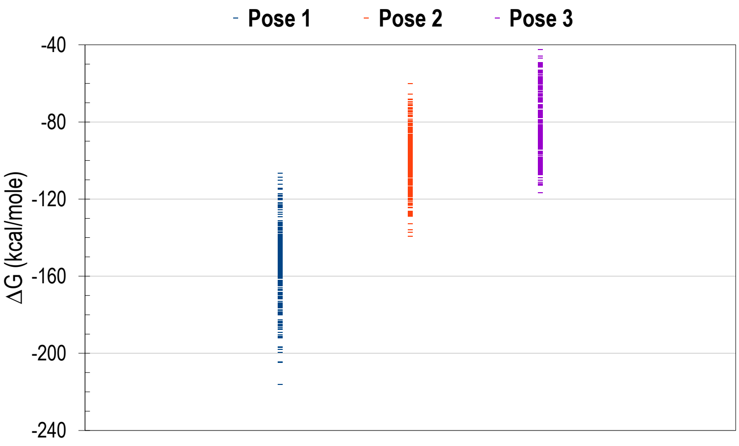

Supplementary Figure 12. Free energy between CALR mutant C-terminus and TpoR D1D2 domain.

Comparative MM-GBSA free energy differences, and their associated standard deviation (SD). Data represent the mean (± SD) of MM-GBSA free energy computed on 100 simulation frames (N = 100) for each pose. The average is -153.43 (± SD 18.49) ΔG (kcal/mol) for pose 1, -100.45 (± SD 14.58) ΔG (kcal/mol) for pose 2 and -81.94 (± SD 17.01) ΔG (kcal/mol) for pose 3.

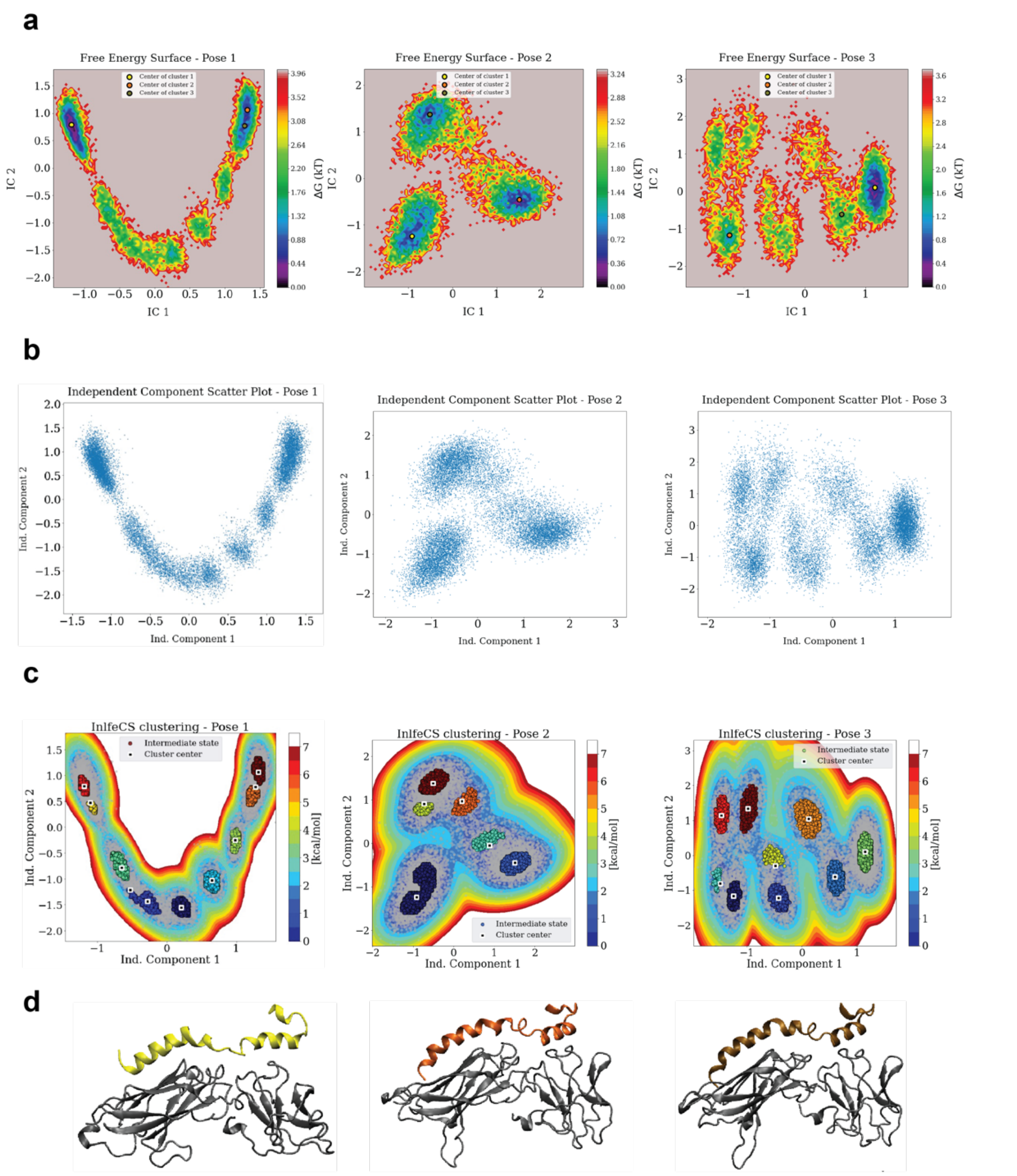

Pose 1, Cluster 1 **Pose 1, Cluster 2** **Pose 1, Cluster 3**

Supplementary Figure 13. *In silico* analyses of the CALR mutant C-terminus interaction with TpoR D1D2 domain.

a. Free energy surface of the independent components for pose 1, pose 2 and pose 3. The centers of the top 3 most probable clusters are shown (see also **Supplementary Methods**). **b.** Top two most auto-correlated components generated by TICA for pose 1, pose 2 and pose 3**. c.** InfleCS cluster of the independent components for pose 1, pose 2 and pose 3. The generated clusters are shown in colored circles and their associated centers in squares. **d.** Cluster center conformation of pose 1. Left: cluster 1, middle: cluster 2, right: cluster 3. These

corresponds to clusters 9, 10 and 8 of **Supplementary Table 1**, respectively. Renders done in VMD v1.9.4.

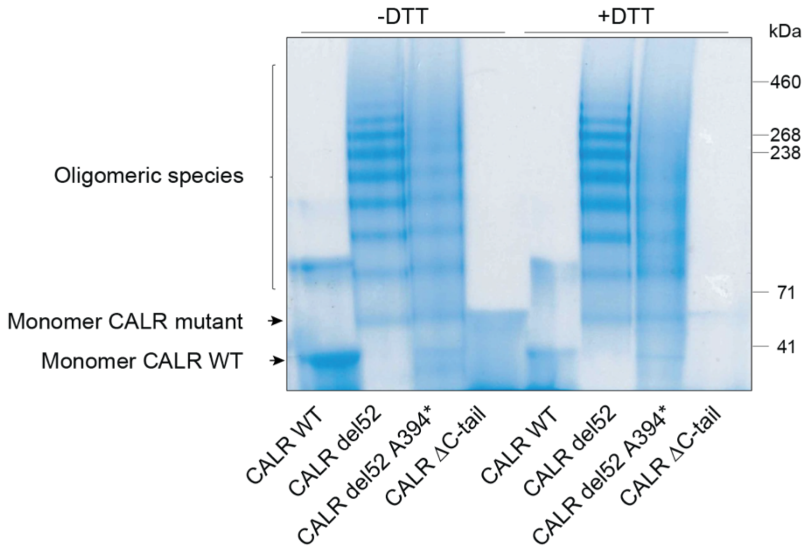

Supplementary Figure 14. CALR mutant oligomerization is independent of C-terminal cysteines.

Native western blot of indicated CALR species, with or without reducing agent (DTT). Staining with Coomassie Blue. Representative gel from 3 experiments.

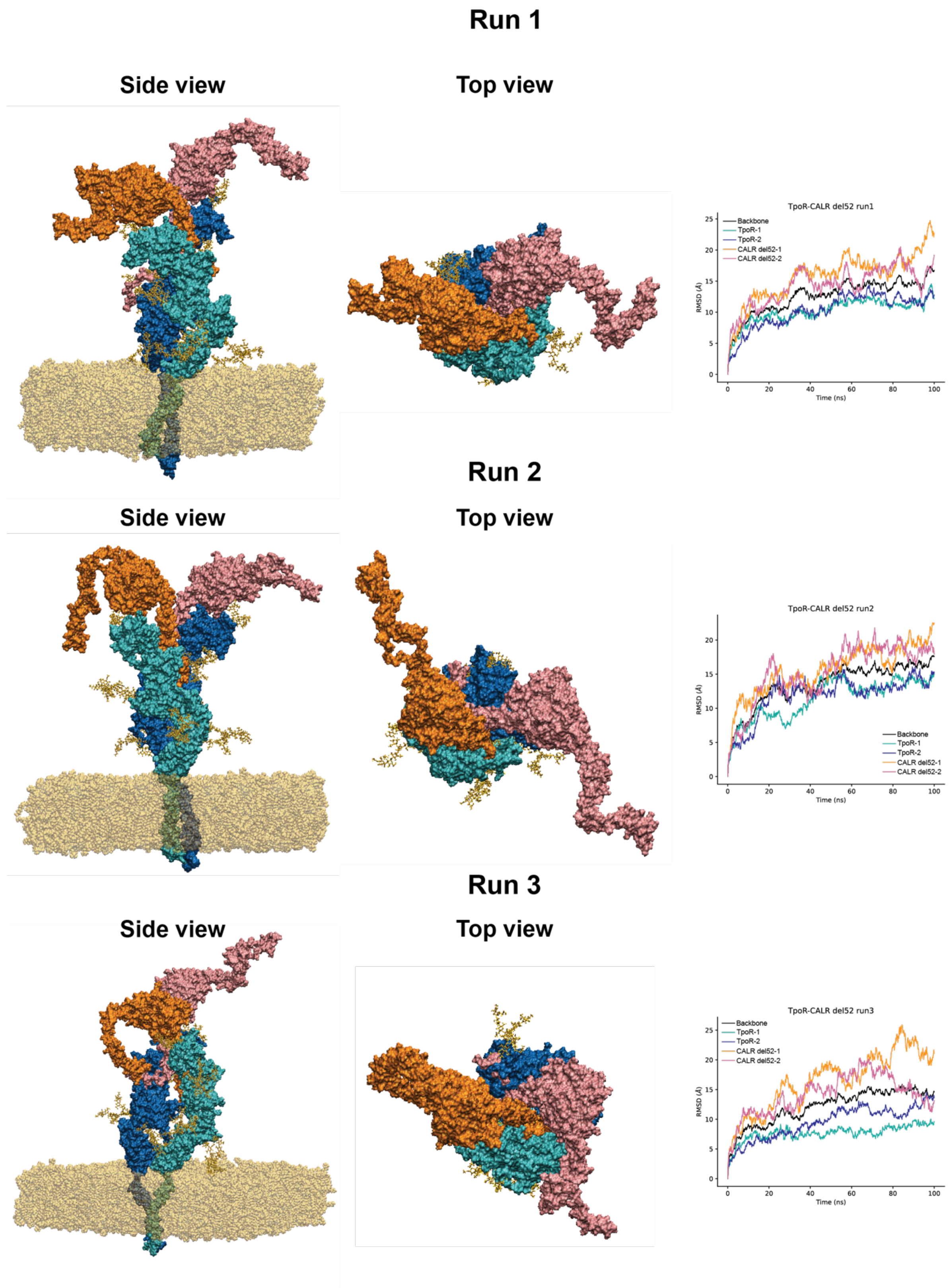

Supplementary Figure 15. Molecular Dynamic Simulations of CALR del52-TpoR tetrameric complex.

Left: side view of the last frame of unconstrained molecular dynamics simulations (100 ns) of the CALR del52-TpoR tetrameric complex performed in triplicates. CALR del52 molecules are shown in orange and pink. TpoR molecules are shown in cyan and dark blue. N-glycans attached to Asn117 (immature), Asn178 (mature), Asn298 (mature) and Asn358 (mature) are shown as yellow sticks. Middle: Top views of the last frame of triplicates molecular dynamics CALR del52-TpoR tetrameric complex. Right: RMSD plots of each molecular dynamic simulations. RMSD over the simulations courses are shown in black (backbone), cyan/light blue (TpoR-1), dark blue (TpoR-2), orange (CALR del52-1) and pink (CALR del52-2).

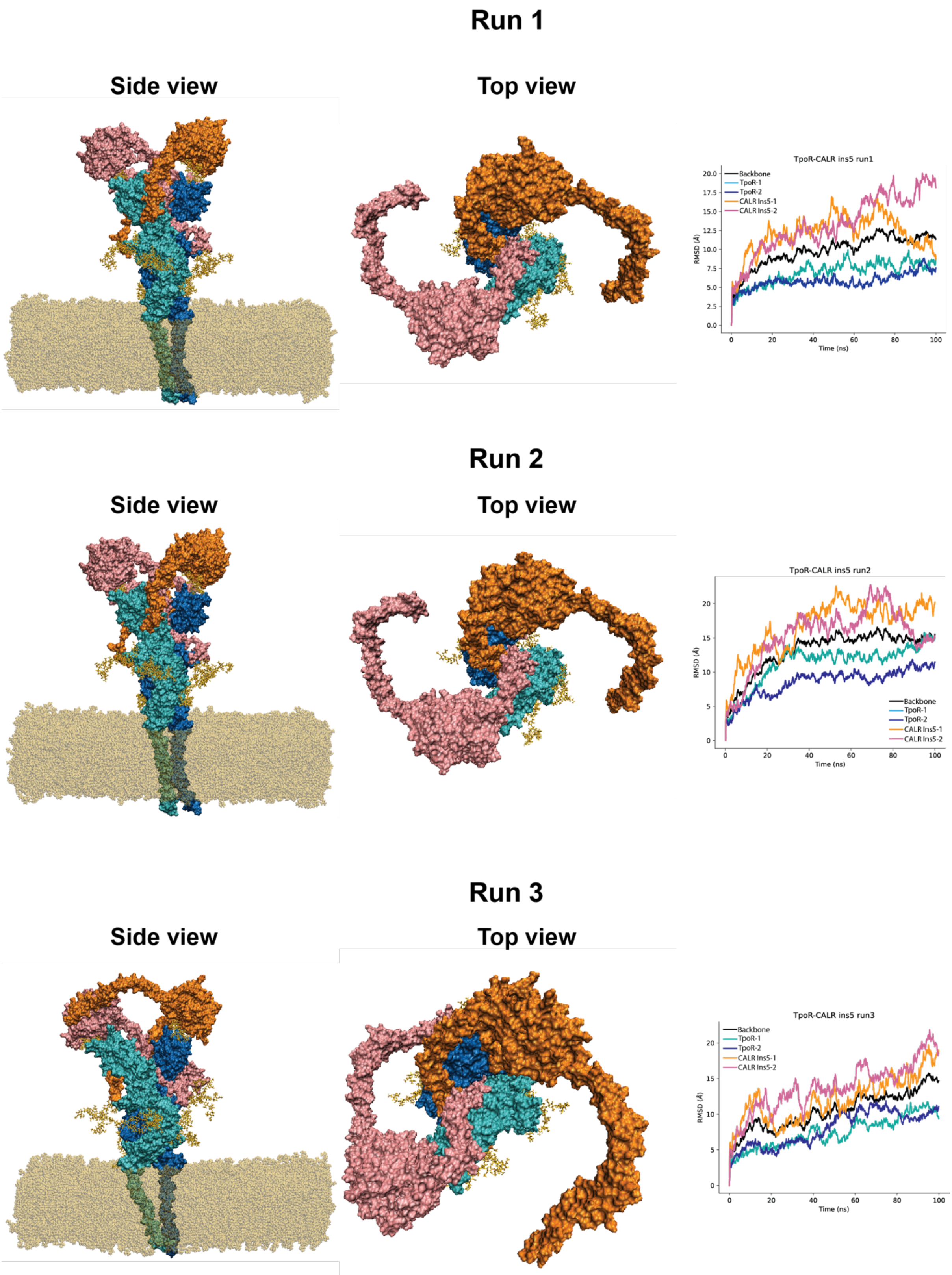

Supplementary Figure 16. Molecular Dynamic Simulations of CALR Ins5-TpoR tetrameric complex.

Left: side view of the last frame of unconstrained molecular dynamics simulations (100 ns) of the CALR Ins5-TpoR tetrameric complex performed in triplicates. CALR Ins5 molecules are

shown in orange and pink. TpoR molecules are shown in cyan and dark blue. N-glycans attached to Asn117 (immature), Asn178 (mature), Asn298 (mature) and Asn358 (mature) are shown as yellow sticks. Middle: Top views of the last frame of triplicates molecular dynamics CALR Ins5-TpoR tetrameric complex. Right: RMSD plots of each molecular dynamic simulations. RMSD over the simulations courses are shown in black (backbone), cyan/light blue (TpoR-1), dark blue (TpoR-2), orange (CALR Ins5-1) and pink (CALR Ins5-2).

Supplementary Table

Cluster Number	State Probability (%)	Cluster Population (determined by Voronoi Tesselation)
1	3%	1441
2	3.2%	886
3	0.05%	595
4	2.3%	955
5	3.3%	1102
6	2.2%	991
7	1.8%	1758
8	4.3%	2035
9	7.6%	3245
10	6.7%	1992

Supplementary Table 1. Detailed overview of the generated InfleCS clusters for pose 1 linked to Supplementary Figure 13d.

Supplementary Methods

Production and purification of recombinant proteins

Recombinant human CALR wild-type, CALR del52 and its derivatives contain a N-terminal His tag sequence (MGSHHHHHHGSSG) that replaces the CALR signal peptide sequence (a.a. 1-17). In addition, the cysteine 163 was mutated to serine. The amino acid sequence of human TpoR D1D2D3D4 (TpoR D1-D4) starts at Q26 and ends at T489 and this of human TpoR D1D2 (TpoR D1D2) starts at Q26 and ends at Q290. Both contains a histidine tag at the C-terminus. The amino acid sequence of human CALR WT starts at E18 and ends at L417.

The sequences of all recombinant proteins are provided below.

Protein	Sequence
**rhCALRWT**	MGSHHHHHHGSSGEPAVYFKEQFLDGDGWTSRWIESKHKSDFGKFVLSSG<br>KFYGDEEKDKGLQTSQDARFYALSASFEPFSNKGQTLVVQFTVKHEQNIDC<br>GGGYVKLFPNSLDQTDMHGDSEYNIMFGPDICGPGTKKVHVIFNYKGKNVLI<br>NKDIRSKDDEFTHLYTLIVRPDNTYEVKIDNSQVESGSLEDDWDFLPPKKIKD<br>PDASKPEDWDERAKIDDPTDSKPEDWDKPEHIPDPDAKKPEDWDEEMDGE<br>WEPPVIQNPEYKGEWKPRQIDNPDYKGTWIHPEIDNPEYSPDPSIYAYDNFG<br>VLGLDLWQVKSGTIFDNFLITNDEAYAEEFGNETWGVTKAAEKQMKDKQDE<br>EQRLKEEEEDKKRKEEEEAEDKEDDEDKDEDEEDEEDKEEDEEEDVPGQA<br>KDEL
**rhCALRdel52**	MGSHHHHHHGSSGEPAVYFKEQFLDGDGWTSRWIESKHKSDFGKFVLSSG<br>KFYGDEEKDKGLQTSQDARFYALSASFEPFSNKGQTLVVQFTVKHEQNIDC<br>GGGYVKLFPNSLDQTDMHGDSEYNIMFGPDICGPGTKKVHVIFNYKGKNVLI<br>NKDIRSKDDEFTHLYTLIVRPDNTYEVKIDNSQVESGSLEDDWDFLPPKKIKD<br>PDASKPEDWDERAKIDDPTDSKPEDWDKPEHIPDPDAKKPEDWDEEMDGE<br>WEPPVIQNPEYKGEWKPRQIDNPDYKGTWIHPEIDNPEYSPDPSIYAYDNFG<br>VLGLDLWQVKSGTIFDNFLITNDEAYAEEFGNETWGVTKAAEKQMKDKQDE<br>EQRTRRMMRTKMRMRRMRRTRRKMRRKMSPARPRTSCREACLQGWTEA
**rhCALRdel52 A394***	MGSHHHHHHGSSGEPAVYFKEQFLDGDGWTSRWIESKHKSDFGKFVLSSG<br>KFYGDEEKDKGLQTSQDARFYALSASFEPFSNKGQTLVVQFTVKHEQNIDC<br>GGGYVKLFPNSLDQTDMHGDSEYNIMFGPDICGPGTKKVHVIFNYKGKNVLI<br>NKDIRSKDDEFTHLYTLIVRPDNTYEVKIDNSQVESGSLEDDWDFLPPKKIKD<br>PDASKPEDWDERAKIDDPTDSKPEDWDKPEHIPDPDAKKPEDWDEEMDGE<br>WEPPVIQNPEYKGEWKPRQIDNPDYKGTWIHPEIDNPEYSPDPSIYAYDNFG

	VLGLDLWQVKSGTIFDNFLITNDEAYAEEFGNETWGVTKAAEKQMKDKQDE EQRTRRMMRTKMRMRRMRRTRRKMRRKMSP
**rhCALR ΔC-tail**	MGSHHHHHHGSSGEPAVYFKEQFLDGDGWTSRWIESKHKSDFGKFVLSSG KFYGDEEKDKGLQTSQDARFYALSASFEPFSNKGQTLVVQFTVKHEQNIDC GGGYVKLFPNSLDQTDMHGDSEYNIMFGPDICGPGTKKVHVIFNYKGKNVLI NKDIRSKDDEFTHLYTLIVRPDNTYEVKIDNSQVESGSLEDDWDFLPPKKIKD PDASKPEDWDERAKIDDPTDSKPEDWDKPEHIPDPDAKKPEDWDEEMDGE WEPPVIQNPEYKGEWKPRQIDNPDYKGTWIHPEIDNPEYSPDPSIYAYDNFG VLGLDLWQVKSGTIFDNFLITNDEAYAEEFGNETWGVTKAAEKQMKDKQDE EQR
**rhCALRdel52 Y109F/D135L**	MGSHHHHHHGSSGEPAVYFKEQFLDGDGWTSRWIESKHKSDFGKFVLSSG KFYGDEEKDKGLQTSQDARFYALSASFEPFSNKGQTLVVQFTVKHEQNIDC GGGFVKLFPNSLDQTDMHGDSEYNIMFGPLICGPGTKKVHVIFNYKGKNVLI NKDIRSKDDEFTHLYTLIVRPDNTYEVKIDNSQVESGSLEDDWDFLPPKKIKD PDASKPEDWDERAKIDDPTDSKPEDWDKPEHIPDPDAKKPEDWDEEMDGE WEPPVIQNPEYKGEWKPRQIDNPDYKGTWIHPEIDNPEYSPDPSIYAYDNFG VLGLDLWQVKSGTIFDNFLITNDEAYAEEFGNETWGVTKAAEKQMKDKQDE EQRTRRMMRTKMRMRRMRRTRRKMRRKMSPARPRTSCREACLQGWTEA
**TpoR D1-D4**	QDVSLLASDSEPLKCFSRTFEDLTCFWDEEEAAPSGTYQLLYAYPREKPRA CPLSSQSMPHFGTRYVCQFPDQEEVRLFFPLHLWVKNVFLNQTRTQRVLFV DSVGLPAPPSIIKAMGGSQPGELQISWEEPAPEISDFLRYELRYGPRDPKNS TGPTVIQLIATETCCPALQRPHSASALDQSPCAQPTMPWQDGPKQTSPSRE ASALTAEGGSCLISGLQPGNSYWLQLRSEPDGISLGGSWGSWSLPVTVDLP GDAVALGLQCFTLDLKNVTCQWQQQDHASSQGFFYHSRARCCPRDRYPIW ENCEEEEKTNPGLQTPQFSRCHFKSRNDSIIHILVEVTTAPGTVHSYLGSPF WIHQAVRLPTPNLHWREISSGHLELEWQHPSSWAAQETCYQLRYTGEGHQ DWKVLEPPLGARGGTLELRPRSRYRLQLRARLNGPTYQGPWSSWSDPTRV ETATETAWHHHHHH
**TpoR D1D2**	QDVSLLASDSEPLKCFSRTFEDLTCFWDEEEAAPSGTYQLLYAYPREKPRA CPLSSQSMPHFGTRYVCQFPDQEEVRLFFPLHLWVKNVFLNQTRTQRVLFV DSVGLPAPPSIIKAMGGSQPGELQISWEEPAPEISDFLRYELRYGPRDPKNS TGPTVIQLIATETCCPALQRPHSASALDQSPCAQPTMPWQDGPKQTSPSRE ASALTAEGGSCLISGLQPGNSYWLQLRSEPDGISLGGSWGSWSLPVTVDLP GDAVALGLQHHHHHH

Production of CALR wild-type and mutants

The corresponding DNA sequences of the CALR recombinant proteins were cloned into a derivative of plasmid pET9 for transformation and expression in the *E. Coli* bacterial strain Rosetta2(DE3). For each production, 2 L of E. Coli culture was centrifuged at 4°C and put on ice. The pellet was lysed in 40 mL buffer A using French press. The lysate was centrifuged at 4.500 G for 20 min. at 4°C and the supernatant was further filtered on 0.45 µM. The clarified lysate was frozen at -80°C until use.

The soluble recombinant proteins were captured from total lysate using Histidine-affinity with a Ni2+ purification resin (HiTRAP IMAC 5mL, Roche) equilibrated in Buffer B. The column was washed with 20 columns volume (100mL) of buffer C. Recombinant proteins were then eluted with by increasing the imidazole concentration (50-200mM). Eluted fractions were concentrated by ultrafiltration and further purified by size-exclusion chromatography using a Superdex 200 Increase 10/300 column (GE healthcare, Chalfont St. Giles, United Kingdom). The proteins were kept in buffer D at -80°C. The size-exclusion chromatographs are provided in **Supplementary Figure 2**. The purity was further confirmed by SDS-PAGE and by measuring the computing the first derivative of the 330/350 nm ratio upon thermal unfolding (**Supplementary Figures 1 and 2**). The correct folding of the proteins was validated by thermal unfolding experiments with Tycho NT.6 (**Supplementary Figure 1**).

Buffer	CALR WT<br>CALR del52 A394*	CALR del52<br>CALR del52 Y109F/D135L	CALR ΔC-tail
**A**	Tween 50mM, NaCl 500mM, CaCl2 5 mM, imidazole 10 mM, CHAPS 1mM, pH 7.5	MES 50mM, NaCl 500mM, CHAPS 1%, EDTA 1mM, pH 6.5	Tween 50mM, NaCl 500mM, CaCl2 5 mM, imidazole 10 mM, CHAPS 1mM, pH 7.5
**B**	Tween 50mM, NaCl 500mM, Imidazole 10 mM, CHAPS 1%, EDTA 1 mM, pH 7.5	MES 50 mM, NaCl 500mM, CHAPS 1%, pH 6.5	Tween 50mM, NaCl 500mM, Imidazole 10 mM, CHAPS 1%, EDTA 1 mM, pH 7.5
**C**	Tween 50mM, NaCl 500mM, Imidazole 10 mM, CHAPS 1%, EDTA 1 mM, pH 7.5	MES 50 mM, NaCl 500mM, CHAPS 1%, imidazole 25 mM (CALR del52) or 10mM (CALR del52 Y109F/D135L), pH 6.5	Tween 50mM, NaCl 500mM, Imidazole 10 mM, CHAPS 1%, EDTA 1 mM, pH 7.5
**D**	HEPES 20mM NaCl 150mM CaCl2 1mM	MES 20mM NaCl 150mM CaCl2 1mM	HEPES 20 mM CaCl2 1mM

Fourrier Transformed Infrared Stepctroscopy (FTIR)

FTIR experiments were performed under the supervision of Allison Derenne, Ph.D., founder and general manager of Spectralys Biotech, Belgium. The full report is provided below.

Sample preparation

In order to avoid any interference with buffers or with the high concentration in salt, a buffer exchange was performed. The buffer was replaced by a phosphate buffer 5 mM at pH 7.01 using Micro-Bio Spin P6 Gel from Bio-Rad. 30µL of the five samples was charged on the columns.

To obtain meaningful comparison, five FTIR spectra were recorded for each sample described here above (after buffer exchange). FTIR spectra (raw data, without any pre-processing step) are provided in **Supplementary Figure 4a.**

Result analysis

To interpret the FTIR data in terms of secondary structure, two types of analysis were performed:

- Comparison of the spectra in the region specific to proteins absorption to evaluate the similarity between the wild type protein and the two mutated forms;
- A prediction of the secondary structure content using an in-house database of FTIR spectra of proteins.

The preprocessing steps described in the methods section are required for meaningful comparison of FTIR spectra. Results shown in main **Figure 1e-f** presents the mean of all preprocessed spectra for each sample with a zoom on the spectral region related to protein absorption.

With the naked eye, clear spectral variations can be noted among the five samples. Regarding the region of the Amide II, it must be noted that the mean spectra of the wild type form is quite different. This would mainly arise from the contribution of the Aspartate and Glutamate residues which are much more abundant in the wild type (55 and 54 respectively) than in CALR del52 (43 and 34), in CALRdel52 A394* (43 and 32), in CALR ΔC-tail (43 and 32) and in CALR del52 D135L/Y109F (42 and 34). These two amino acids have a significant contribution around 1568 cm^{-1} due to the absorption of the carbonyl group in their side chains.

In addition to this first observation, it can be noticed that the CALR WT and the CALRdel52 A394* have a similar shape, especially in the amide I band. The CALR ΔC-tail has a similar shape in the amide I band but exposes very different characteristics in the amide II band. The CALRdel52 and the CALRdel52 D135L/Y109F have each, specific and distinct features in both, the amide I and the amide II bands.

Multivariate analyses can be applied to assess similarities and discrepancies among the samples. Principal component analysis (PCA) is a powerful tool for high-dimension data to identify patterns and to express the data in such a way as to highlight their similarities and differences. This tool reduces the number of variables without much loss of information, taking into account most of the variance. It is an unsupervised analysis: the classification obtained does not suppose any a priori condition on grouping obtained. Technical details concerning PCA are provided in the "Methods" section.

Supplementary Figure 4b displays the PCA score plot. Every point (star) in this plot is the projection of one spectrum in the space defined by the first two principal components (PC). The different samples are identified by a unique colour (indicated in the right caption). Each mutant clusters separately, underlying that each mutant has specific spectral features.

The CALR del52 A394* and the CALR del52 separate from the CALR WT along the first principal component (PC1). The CALR del52 A394* is the closest to the CALR WT. The two other mutants separate from the CALR WT along the PC1 and the PC2.

Secondary structure prediction

As described in the methods section, the estimation was realized using three wavenumbers in the Amide I and II bands. The wavenumbers used for this secondary structure determination are the following:

- α-helix: 1545, 1655 and 1613 cm^{-1}
- β-sheet: 1656, 1635 and 1692 cm^{-1}
- Turn: 1678, 1528 and 1600 cm^{-1}
- Random: 1544, 1627 and 1692 cm^{-1}

The table shown in main **Figure 1f** presents the results of this prediction for each sample.

According to the prediction obtained, samples CALR WT and CALR del52 A394* have a similar α-helix, β-sheet, turn and random structures content. Sample CALR del52 has a lower content in α-helix and a slightly higher content in random structures. The α-helix content further decreases for sample CALRdel52 D135L/Y109F and becomes null for CALR ΔC-tail. In addition, the CALR del52 D135L/Y109F has a higher content in turn and the CALR ΔC-tail has a higher turn and random contents. A slight increase of the β-sheet content is also observed for the CALR ΔC-tail.

For the present predictions, the standard error of prediction in cross-validation obtained using the 50-protein database is 5.7% for the α-helix and 6.7% for the β-sheet, 3.2% for turns and 8% for random. Despite these prediction errors, those results are consistent with the observations made on main **Figure 1e** and **Supplementary Figure 4a.**

<u>Instrument</u>

The IR measurements were performed with a Bruker Tensor 27 FTIR-spectrometer (Bruker Optics GmbH, Ettlingen, Germany) with the software Opus 6.5 (Bruker Optics GmbH, Ettlingen, Germany). The FTIR-spectrometer was equipped with a Mercury-Cadmium-Telluride detector, which was cooled down with liquid nitrogen. The spectra were recorded with the ATR mode by using a Golden GateTM ATR accessory (Specac, Orpington, United Kingdom) with an integrated total reflection element composed of a single reflection diamond. The angle of incidence was 45 degrees.

<u>Methods</u>

FTIR measurement

0.5 µL of sample was loaded on the diamond crystal of the ATR device of the FTIR spectrometer and quickly dried with a constant, gentle nitrogen flow: elimination of the water molecules prevents overlapping of the large water absorption peaks with the sample's absorption spectrum. After each spectrum, the crystal was cleaned with water. A background was recorded with a clean crystal before the start of the measurement and before every new sample. FTIR spectra were recorded between 4000 and 600 cm^{-1} at a resolution of 2 cm^{-1}. Each spectrum was obtained by taking an

average of 128 scans. The FTIR measurements were carried out at room temperature (~22°C). For each sample, at least four spectra were recorded.

Multivariate data analysis

Each wavelength in an IR spectrum is considered as a variable. There are therefore a few thousand wavenumbers at which biological molecules absorb, and several spectra are recorded per sample. Thus, the measured data contains a high number of variables, which are additionally often correlated with each other. Consequently, the analysis and interpretation of this large amount of information is complicated. To extract useful information from the measured data, multivariate data analysis can be applied. Multivariate data analysis can be carried out based on unsupervised or supervised learning procedures. For the unsupervised procedures no a priori knowledge about the training set samples is required.

Principal component analysis (PCA) is an unsupervised multivariate data analysis. This technique permits reducing the dimensionality of the data consisting of many correlated variables. At the same time, most of the variation present in the data set is retained [3]. Therefore the variables are transformed in a new uncorrelated set of variables, the principal components (PC) [3]. The PC are linear combinations of the initial variables representing a maximum of variation present in the data [3] . This transformation simplifies the interpretation and visualisation of the data [4].

Mathematically, each IR spectrum can be represented as a linear combination of p wavenumbers. For n spectra the matrix A [nxp] (samples x variables) can be expressed whereat one spectrum corresponds to one row and each column characterises one wavenumber. Thus, each element of the matrix represents the absorbance of a spectrum at one specific wavenumber. The principal components are determined by calculating the eigenvalues and their corresponding eigenvectors of the covariance matrix A [pxp’]. In the next step, the eigenvectors are ordered by eigenvalue, from the highest one to the lowest one, to obtain the principal components in order of their significance. The eigenvector with the largest eigenvalue is defined as the first principal component (PC1) as it represents the direction of the greatest variance. The eigenvector with the second largest eigenvalue (PC2) represents the next highest variance in the orthogonal direction and so forth. Finally, the coordinate system of the original spectral data will be transformed to express the spectral data in

terms of the principal components as new axes. This transformation is demonstrated in **Supplementary Figure 4c**. In fact, the plot of the original data in terms of the axes x and y will be rotated and the principal components form the new axes.

In fact, there are as many principal components as variables in the data. However, the first few principal components represent generally over 99% of the present variance in the data. Thus, PCA permits reducing the dimensionality of the spectral data while retaining the majority of the information. This is simply done by projecting the spectra in the principal components space.

The representation of the composition of all spectra in terms of the PC is called score plot. Each point or star in a score plot represents a spectrum. Thus, a score plot permits visualising similarities and difference between spectra and to determine if the spectra are related with each other by forming groups [5].

Secondary structure prediction

Using a database of 50 protein containing as little fold redundancy as possible, an ascending stepwise method was applied to determine the protein secondary structure. It was demonstrated that three wavenumbers contain all the nonredundant information related to the secondary structure content. The standard error of prediction in cross-validation obtained using the 50-protein database was 5,7% for the α-helix and 6,7% for the β-sheet, 3.2% for turns and 8% for random [6,7].

Statistical analysis

Supplementary Figure 5 presents the mean preprocessed spectra for each sample, with a zoom on the spectral region related to protein absorption. A statistical pairwise comparison with the wild type was also performed to evidence spectral changes. The difference spectrum (black spectrum) corresponds to the difference between the mean spectra of each sample. The black stars on the difference spectrum refer to significant differences defined by a Student's t-test at each wavenumber. Details on the statistical techniques are provided here below.

<u>Student's t-test</u>

In order to evidence spectral changes between samples, the mean spectrum of one was subtracted from the mean spectrum of another. We thus obtained a "difference

spectrum". All difference spectra were calculated with fully preprocessed spectra (baseline corrected and normalized).

The Student's t-test is a parametrical hypothesis test. It is used to determine whether two populations are significantly different from each other or not by comparing the means of the measurements derived from these two populations. The test is applicable if the measurements follow a normal distribution, and the variance of each population is the same.

Two hypotheses are tested:

- H_0: $\lambda_1 = \lambda_2$ which means that there is no difference between the means of the two populations
- H_1: $\lambda_1 \neq \lambda_2$ which means that the means of the two populations are significantly different

where λ_i is the signal intensity for a given wavelength.

The t-test statistic is calculated as follows:

$$t = \frac{(\bar{x}_1) - (\bar{x}_2)}{\sqrt{\frac{S_P^2}{n_1} + \frac{S_P^2}{n_2}}}$$

where $\bar{x}_i$ is the mean of the sample i, S_P^2 is the estimated common variance for the two samples and n_i the number of the sample in the population i.

The variance S_P^2 is calculated with the following formula:

$$S_P^2 = \frac{(n_1 - 1)s_1^2 + (n_2 - 1)s_2^2}{n_1 + n_2 - 2}$$

where s_i is the standard deviation of the sample i and n_i the number of the samples in the population i.

The test was carried out with a significance level of $\alpha = 0.1\%$ ($p<0.001$). This threshold is defined as the probability of rejecting the null hypothesis under the assumption that it is true.

Student's t-tests were computed at every wavenumber and allowed a statistical comparison between the spectra of the two samples. Wavenumbers where a significant difference occurs (with a significance $\alpha = 0.1\%$) are indicated by black stars.

Nano-Bioluminescence Energy Transfer (BRET)

Nano-Bioluminescence Energy Transfer (BRET) is a technique that measures proximity between two proteins in living cells. When the two partners are in close proximity (< 10 nm), bioluminescence energy transfer (BRET) occurs between a donor (NanoLuciferase) and an acceptor (HaloTag ligand. This technique has been used to measure protein-protein interaction in living cells between a wide variety of proteins thanks to its ease of use, reproducibility and specificity [8].

Construct

The Thrombopoietin Receptor (TpoR) was cloned into pNL-N vector (Promega) to generate the N-terminally fused NanoLuc-TpoR construct as described. Extracellular forms of the receptor (TpoR D1-D4, TpoR D1D2 and TpoR D1) were obtained from this initial construct by introducing a stop codon by site-directed mutagenesis.

The CALR del52-HaloTag construct was generated by cloning the cDNA from CALR del52 into the pHT-C vector (Promega) to generation the CALR del52-HaloTag fusion protein[9]. The CALR del52 P-C and C-domain-HaloTag were obtained by truncating the N domain or N and P-domain, respectively using the Q5® Site-Directed Mutagenesis method with KLD (NEB). All constructions were sequenced by Macrogen.

Methods

HEK293T were co-transfected with the NanoLuc and HaloTag fusion proteins with a 1:1 cDNA ratio using TransLT-1 in white opaque 96-wells plate. The NanoBRET 618 ligand was added 8h post-transfection according to the manufacturer instruction (Promega). 24h post-transfection, the medium was removed and 100 µL of luminescence substrate (DMEM/F12 without FBS, without phenol red with 2 µL of NanoGlo reagent) was added to each well. The signal was acquired directly using a GloMax® MicroPlate reader at 618 nm (acceptor) and 460 nm (donor) at 37°C. The NanoBRET ratio was computed as:

$$\frac{\text{Acceptor signal}}{\text{Donor Signal}} = \text{NanoBRET ratio}$$

The specificity of the interaction was further validated by performing a Donor Saturation Assay (DSA) (**Supplementary Figure 7**) following the manufacturer instruction (Promega).

Molecular Modelling, Docking, Molecular Dynamics and Free energy simulations

Generation of TpoR D1D2 and CALR mutant C-terminus

Sequences of TpoR extracellular regions which consists of two Cytokine Receptor Modules (CRM1 and 2) comprising each two Fibronectin III like (FN-III) domains and of CALR Del52 were profiled for secondary structure, intrinsic disorder and accessibility propensities with state-of-the-art predictors [10-20]. Models were raised manually for CRM1 using Modeller 9.21 [17], and automatically for the TpoR-CRM2, TpoR-linker, TpoR-Transmembrane domain and CALR using Alpha Fold 2.0 [1] and Rosetta Folding [1].

When compared to its closest template (EpoR, RCSB code: 1CN4) - TpoR displays a long, 65 amino acids insertion in its second FN-III domain of CRM1 which bears local propensity for two ß-strands. Hence a second template, the 2E8 antibody of LDL receptor (RCSB code: 12E8) from the same CATH 2.60.40.10 superfamily, has had to be used to model the insertion and bring the TpoR-CRM1 architecture from a 3/4 β-sandwich (EpoR) to a 4/5 β-sandwich TpoR model.

In order to computationally investigate the interaction between CRM1 and CALR-del52, a model of the mutant C-terminus of CALR del52 was also generated using tLEaP and the FF14SB [21] protein forcefield. Given the secondary structure propensity of CALR del52 C-terminus, this region was modelled first as a straight, ~72Å long, 12 turn α-helix. This structure was then subjected to a 1 µs MD simulation in order to gather the conformational pool to be used in the selection of several starting CALR del52 mutant C-terminus structures for CALR del52 C-terminus-TpoR D1D2 docking. The simulation was performed with OpenMM v.7.4.1 [22] in explicit solvent, using a Monte Carlo barostat to maintain constant pressure, at a temperature of 300K. The conformation clustering along the trajectory and structure extraction was performed with TASKit, an in-house developed Python module, based on the All-vs-All RMSD matrix for clustering and on MDTraj [23] and SciPy [24] for structure extraction. Using a maximum inter-cluster distance cutoff of 8Å, two major configurational basins were identified, from which structural selections were performed. The structures in these

two basins differ by the bending angle of a break in the α-helix shaping up at the 'PARP' motif of CALR del52 (**Supplementary Figure 10a**).

Docking trials between TpoR D1D2 and CALR del52 mutant C-terminus

Taking into account the location of negatively charged acidic amino acids onto the surface of TpoR, three main start configurations (*poses*) of the complex were chosen for assessing the complex formation: one involving a potential interaction of CALR del52 mutant C-terminus with the large extended acidic area found on the 'dorsal' region of TpoR, a second similar pose - but using the more bent CALR del52 mutant C-terminus configuration, and a third involving the second, minor acidic cluster found in the N-ter region of the TpoR sequence. Several structures from the two configurational basins of CALR-del52 were manually set in the above-mentioned poses of the complex as inputs for HADDOCK 2. 4 [8] for complex optimization searches (**Supplementary Figure 10b**). The positively charged, basic residues on CALR and negatively charged, acidic residues on TpoR were selected as “interacting residues” in the HADDOCK run and no other explicit restraints were used. The generated poses were then filtered by their HADDOCK score and the top three poses were chosen for further analysis.

Molecular dynamics simulations of TpoR D1D2-CALR del52 mutant C-terminus complex and subsequent analysis

Starting from the three poses generated by HADDOCK, we sampled the conformational space of the complex via unconstrained molecular dynamics simulations. Each of the three poses were subjected to triplicate 500ns molecular dynamics simulations in which the last residues of the CALR del52 mutant C-terminus become unfolded, in explicit TIP3P water boxes with a 12Å buffer, using a Monte Carlo barostat, at 300K, a Langevin integrator with 1ps^{-1} friction coefficient and a 2fs timestep. OpenMM [22] was used to run all simulations, with the same ff14SB [21] forcefield. PDB files corresponding to the first and last frame of triplicates MD simulations for each pose have been deposited to FigShare (https://figshare.com/s/9033970b5a1d3f8d6fa7).

Trajectory analysis

A strong bending motion was noticed in the TpoR molecule of Pose 3 (lowest binding free energy) between the two FN-III-like domains (**Supplementary Figure 10c-d**). This was not observed in the other poses (**Supplementary Figure 10e-f**) which could

indicate that the presence of the CALR del52 C-terminus has a stabilizing effect on the TpoR. This might also imply that the domain is more flexible when unbound, but the inter domain joint stiffens when bound.

Free energy estimations

Free energy was estimated by both a knowledge based method, using PRODIGY server[25], and a physical MD estimation approach based on 3 simulations for each pose, using the MM-GBSA method [26] at 150mM salt concentration, implemented in AMBER20 [27]. Results using both methods were in line with HADDOCK scores.

Free energy estimation using the PRODIGY: the best free energy is shown by the dark-blue complex (**Supplementary Figure 10b**) with ΔG= –12.6 kcal/mol, the sky-blue complex displays an intermediate estimated affinity with ΔG= –11.2 kcal/mol, while the red complex shows the lowest binding capacity with ΔG= –9.6 kcal/mol.

*Free energy estimation using MM-GBSA***:** The first half (250ns) of simulation time was removed as equilibration and 100 frames were uniformly drawn at every 2.5ns, from each repeat, and retained for MM-GBSA calculation. MM-GBSA calculations were performed using the MM-PBSA module included with AMBER20 [28]. A total of 300 frames were used for each pose. The OBC GB model [29] and a salt concentration of 150 mM was used. The LCPO algorithm [30] was used to calculate the surface area of the solute.

The three poses' binding energy estimates using MM-GBSA show exactly the same trend as initial PRODIGY estimates and provide insight into the relative stabilities of the three poses. The values presented in **Supplementary Figure 12** show that the stretched CALR del52 C-terminus conformation (Pose 1) *binds the strongest* to the membrane-distal TpoR domain and that Pose 3 binds the weakest. This could explain the higher flexibility of the TpoR domain when bound in the Pose 3 configuration.

Conformational discretization:

Microstates were delimited using Time-Lagged Independent Component Analysis (TICA). The backbone dihedral angles of the CALR del52 mutant C-terminus molecule were used as input coordinates for TICA. TICA and free energy surfaces were computed using the PyEMMA (2.5.11) python package[31], and the resulting plots were generated using the Matplotlib (3.5.1) python package [32].

The inflection core state (InfleCS) clustering method [33] was used to cluster the two transformed coordinates with the highest eigenvalues and the associated cluster centers were plotted on the corresponding free energy surface. Clustering was performed using 10 components, and re-estimation of the same model was done 5 times. Bayesian information criterion was used for identifying the model.

Trajectory clustering using TICA

The independent components generated by TICA were sorted by their eigenvalues and the top two most autocorrelated components were plotted (**Supplementary Figure 13b**). The subsequent InfleCS clustering (**Supplementary Figure 13c**, **Supplementary Table 1**) shows the formation of discrete conformational clusters, showing that a range of microstates were sampled. Free energy surfaces were generated and are presented in **Supplementary Figure 13a**, along with the centers of the top 3 most probable clusters for pose 1, for which trajectory frames are also presented (**Supplementary Figure 13d**).

Modelling of the TpoR-CALR del52 and ins5 tetramers

Initial models from AlphaFold 2.0[1] and RosettaDock[34] were used to build the tetrameric 3D TpoR-CALR mutant models and to identify the interaction interface between the two CALR del52 and CALR ins5 mutants. H-D exchange data was used to identify contacts between TpoR and CALR del52 in the formation of the tetramer complex. The ER specific G1M9 glycans of TpoR in contact to CALR mutant were modelled with Glycopack [35] and the Glycam server in the configuration consistent with NMR data [36] while the rest are of complex type, built in agreement with SAGS Database [37,38].

The contacts identified by HDx-MS and the crosslinking data on the TM region configuration of TpoR dimer were used as constraints in generating the overall 2CALR del52-2TpoR model. The glycoproteic tetramer was then gently optimized in a five stage process in implicit solvent: (1) first, this was heated to 300K over 1 ns with harmonic cartesian constraints of K=1 on all backbone atoms predicted to be found in secondary structures and K=0.5 on backbone atoms in predicted coil regions; then (2) the system was subjected to equilibration for 2 ns with harmonic cartesian constraints of K=0.5 on secondary structures and no constraints on predicted coil region; this was followed by (3) a further equilibration of 20ns with distance based harmonic constraints of K=1 on interdomain contact points and hydrogen bonds in predicted secondary

structure regions; then (4) the system was cooled over 2 ns with distance based constraints in place and finally (5) extensively minimized without constraints.

This glycoproteic tetramer was then immersed into a full-atom representation of the environment - consisting of a lipid bilayer of 1162 POPC molecules accommodating the TM region of TpoR and in 263023 TIP3P water molecules, 726 chloride and 789 sodium ions describing the solvent region hydrating the rest of the tetramer using the CHARMM-GUI server [39]. This overall system consisting of ~ 1 million atoms was subjected to further unconstrained extensive minimization to obtain the final model for MD simulation. A similar procedure was used for preparing the system containing the TpoR-CALR ins5 tetramer.

The explicit solvent MD simulations were performed with NAMD v.2.13[36] and the CHARMM36 [37-39] forcefield. at constant pressure (1 atm) in two steps: (1) heating using a 1fs time step in order to ensure an even energy distribution, followed by (2) constant temperature simulation using a 2 fs timestep. All MD simulations used a Langevin integrator, with a coupling coefficient of 1 ps^{-1}.

Production MD simulations of the heterotetrameric TpoR-CALR del52 and ins5 systems were carried out for 100 ns in triplicate. Root-mean-square deviation (RMSD) analysis of these simulation was performed using MDAnalysis [41,42] and the corresponding plots are presented in Supplementary Figure 8 (CALR del52) and Supplementary Figure 9 (CALR Ins5). For the analysis of inter-residues contacts, the first half (50 ns) of each simulation was discarded as equilibration and only the second half (which shows a plateau in the RMSD plot) was used for computing the distances.

Tetramer trajectory analysis

Inter-domain and glycan-CALR contacts were computed using MDTraj[19] and were averaged over three 100ns runs. A threshold of 8 Å was used as contact cutoff between heavy atoms and the data was collected every 0.5 ns starting from 50 ns - to account for a model equilibration period – resulting in 100 frames per run. Shown are only contacts present, on average, in more than 60% of frames.

Thermal unfolding and stability

Thermal unfolding experiments were conducted to measure the T_m of all proteins used in HDx-MS and FTIR experiments and to verify the quality and purity of protein preparations. This technique uses the intrinsic fluorescence of Trp and Tyr residues to verify quality, purity and structural integrity of a protein. The intrinsic fluorescence of Trp and Tyr residues (detected at 350 and 330 nm) are measured over a range of increasing temperature. As temperature ramps up, changes in fluorescence signal inform on the protein folding status. The 350/350 nm ratio provides information about protein integrity and T_m. The first derivative of the 350/330 ratio informs on the purity of the protein preparation. A pure sample will have a single peak, indicating that a single protein specie is present in the sample while samples that are not pure will have multiple peaks. All measurements were performed with Tycho NT.6 (NanoTemper).

Supplementary References

1. Jumper, J. et al. Highly accurate protein structure prediction with AlphaFold. *Nature* (2021).
2. Lau, A.M., Claesen, J., Hansen, K. & Politis, A. Deuteros 2.0: peptide-level significance testing of data from hydrogen deuterium exchange mass spectrometry. *Bioinformatics* **37**, 270-272 (2021).
3. Jolliffe, I.T. *Principal component analysis for special types of data*, (Springer, 2002).
4. Ringnér, M. What is principal component analysis? *Nature Biotechnology* **26**, 303-304 (2008).
5. Boydston-White, S. et al. Cell-cycle-dependent variations in FTIR micro-spectra of single proliferating HeLa cells: principal component and artificial neural network analysis. *Biochim Biophys Acta* **1758**, 908-14 (2006).
6. Goormaghtigh, E., Ruysschaert, J.M. & Raussens, V. Evaluation of the information content in infrared spectra for protein secondary structure determination. *Biophys J* **90**, 2946-57 (2006).
7. Oberg, K.A., Ruysschaert, J.M. & Goormaghtigh, E. Rationally selected basis proteins: a new approach to selecting proteins for spectroscopic secondary structure analysis. *Protein Sci* **12**, 2015-31 (2003).
8. Dale, N.C., Johnstone, E.K.M., White, C.W. & Pfleger, K.D.G. NanoBRET: The Bright Future of Proximity-Based Assays. *Frontiers in Bioengineering and Biotechnology* **7**(2019).
9. Pecquet, C. et al. Calreticulin mutants as oncogenic rogue chaperones for TpoR and traffic-defective pathogenic TpoR mutants. *Blood* **133**, 2669-2681 (2019).
10. Barik, A. et al. DEPICTER: Intrinsic Disorder and Disorder Function Prediction Server. *J Mol Biol* **432**, 3379-3387 (2020).
11. Dominguez, C., Boelens, R. & Bonvin, A.M. HADDOCK: a protein-protein docking approach based on biochemical or biophysical information. *J Am Chem Soc* **125**, 1731-7 (2003).
12. Ishida, T. & Kinoshita, K. PrDOS: prediction of disordered protein regions from amino acid sequence. *Nucleic Acids Res* **35**, W460-4 (2007).
13. Kelley, L.A., Mezulis, S., Yates, C.M., Wass, M.N. & Sternberg, M.J. The Phyre2 web portal for protein modeling, prediction and analysis. *Nat Protoc* **10**, 845-58 (2015).
14. Petersen, B., Petersen, T.N., Andersen, P., Nielsen, M. & Lundegaard, C. A generic method for assignment of reliability scores applied to solvent accessibility predictions. *BMC Structural Biology* **9**, 51 (2009).
15. Romero, P. et al. Sequence complexity of disordered protein. *Proteins* **42**, 38-48 (2001).
16. Rost, B. & Sander, C. Prediction of protein secondary structure at better than 70% accuracy. *J Mol Biol* **232**, 584-99 (1993).
17. Webb, B. & Sali, A. Comparative Protein Structure Modeling Using MODELLER. *Curr Protoc Bioinformatics* **54**, 5.6.1-5.6.37 (2016).
18. Drozdetskiy, A., Cole, C., Procter, J. & Barton, G.J. JPred4: a protein secondary structure prediction server. *Nucleic Acids Research* **43**, W389-W394 (2015).
19. Jones, D.T. Protein secondary structure prediction based on position-specific scoring matrices. *J Mol Biol* **292**, 195-202 (1999).
20. Yachdav, G. et al. PredictProtein—an open resource for online prediction of protein structural and functional features. *Nucleic Acids Research* **42**, W337-W343 (2014).

21. Maier, J.A. et al. ff14SB: Improving the Accuracy of Protein Side Chain and Backbone Parameters from ff99SB. *J Chem Theory Comput* **11**, 3696-713 (2015).
22. Eastman, P. et al. OpenMM 7: Rapid development of high performance algorithms for molecular dynamics. *PLoS Comput Biol* **13**, e1005659 (2017).
23. McGibbon, R.T. et al. MDTraj: A Modern Open Library for the Analysis of Molecular Dynamics Trajectories. *Biophys J* **109**, 1528-32 (2015).
24. Virtanen, P. et al. SciPy 1.0: fundamental algorithms for scientific computing in Python. *Nature Methods* **17**, 261-272 (2020).
25. Xue, L.C., Rodrigues, J.P., Kastritis, P.L., Bonvin, A.M. & Vangone, A. PRODIGY: a web server for predicting the binding affinity of protein-protein complexes. *Bioinformatics* **32**, 3676-3678 (2016).
26. Kollman, P.A. et al. Calculating structures and free energies of complex molecules: combining molecular mechanics and continuum models. *Acc Chem Res* **33**, 889-97 (2000).
27. D.A. Case, H.M.A., K. Belfon, I.Y. Ben-Shalom, J.T. Berryman, S.R. Brozell, D.S. Cerutti, T.E. Cheatham, III, G.A. Cisneros, V.W.D. Cruzeiro, T.A. Darden, R.E. Duke, G. Giambasu, M.K. Gilson, H. Gohlke, A.W. Goetz, R. Harris, S. Izadi, S.A. Izmailov, K. Kasavajhala, M.C. Kaymak, E. King, A. Kovalenko, T. Kurtzman, T.S. Lee, S. LeGrand, P. Li, C. Lin, J. Liu, T. Luchko, R. Luo, M. Machado, V. Man, M. Manathunga, K.M. Merz, Y. Miao, O. Mikhailovskii, G. Monard, H. Nguyen, K.A. O'Hearn, A. Onufriev, F. Pan, S. Pantano, R. Qi, A. Rahnamoun, D.R. Roe, A. Roitberg, C. Sagui, S. Schott-Verdugo, A. Shajan, J. Shen, C.L. Simmerling, N.R. Skrynnikov, J. Smith, J. Swails, R.C. Walker, J. Wang, J. Wang, H. Wei, R.M. Wolf, X. Wu, Y. Xiong, Y. Xue, D.M. York, S. Zhao, and P.A. Kollman AMBER 2020. (University of California, San Francisco, 2020).
28. Miller, B.R. et al. MMPBSA.py: An Efficient Program for End-State Free Energy Calculations. *Journal of Chemical Theory and Computation* **8**, 3314-3321 (2012).
29. Onufriev, A., Bashford, D. & Case, D.A. Exploring protein native states and large-scale conformational changes with a modified generalized born model. *Proteins: Structure, Function, and Bioinformatics* **55**, 383-394 (2004).
30. Weiser, J., Shenkin, P.S. & Still, W.C. Approximate atomic surfaces from linear combinations of pairwise overlaps (LCPO). *Journal of Computational Chemistry* **20**, 217-230 (1999).
31. Scherer, M.K. et al. PyEMMA 2: A Software Package for Estimation, Validation, and Analysis of Markov Models. *Journal of Chemical Theory and Computation* **11**, 5525-5542 (2015).
32. Hunter, J.D. Matplotlib: A 2D Graphics Environment. *Computing in Science & Engineering* **9**, 90-95 (2007).
33. Westerlund, A.M. & Delemotte, L. InfleCS: Clustering Free Energy Landscapes with Gaussian Mixtures. *Journal of Chemical Theory and Computation* **15**, 6752-6759 (2019).
34. Lyskov, S. & Gray, J.J. The RosettaDock server for local protein-protein docking. *Nucleic Acids Res* **36**, W233-8 (2008).
35. Paduraru, C. et al. An N-linked glycan modulates the interaction between the CD1d heavy chain and beta 2-microglobulin. *J Biol Chem* **281**, 40369-78 (2006).
36. Petrescu, A.J. et al. The solution NMR structure of glucosylated N-glycans involved in the early stages of glycoprotein biosynthesis and folding. *Embo j* **16**, 4302-10 (1997).

37. Petrescu, A.J., Petrescu, S.M., Dwek, R.A. & Wormald, M.R. A statistical analysis of N- and O-glycan linkage conformations from crystallographic data. *Glycobiology* **9**, 343-52 (1999).
38. Petrescu, A.J., Wormald, M.R. & Dwek, R.A. Structural aspects of glycomes with a focus on N-glycosylation and glycoprotein folding. *Curr Opin Struct Biol* **16**, 600-7 (2006).
39. Jo, S., Kim, T., Iyer, V.G. & Im, W. CHARMM-GUI: A web-based graphical user interface for CHARMM. *Journal of Computational Chemistry* **29**, 1859-1865 (2008).
