# Supplementary material for: Oncogenic CALR mutant C-terminus mediates dual binding to the thrombopoietin receptor triggering complex dimerization and activation
Source: Nat Commun. 2023 Apr 5;14:1881. doi: 10.1038/s41467-023-37277-3 (PMC10076285; doi:10.1038/s41467-023-37277-3)
Supplement: Supplementary file 2 — Description of Additional Supplementary Files [file 41467_2023_37277_MOESM2_ESM.docx]

**Description of Supplementary Data files**

**Filename: Supplementary Data 1**

**Description**: Contacts between CALR del52 and TpoR during triplicate 100ns MD runs of the CALR del52-TpoR tetrameric complex. Contacts are defined as residues with a distance < 8Ä. Only contacts present in >60% of frames (average of triplicates) are shown.

**Filename: Supplementary Data 2**

**Description**: Contacts between CALR Ins5 and TpoR during triplicate 100ns MD runs of the CALR Ins5-TpoR tetrameric complex. Contacts are defined as residues with a distance < 8Ä. Only contacts present in >60% of frames (average of triplicates) are shown.
