# Supplementary material for: Oncogenic CALR mutant C-terminus mediates dual binding to the thrombopoietin receptor triggering complex dimerization and activation
Source: Nat Commun. 2023 Apr 5;14:1881. doi: 10.1038/s41467-023-37277-3 (PMC10076285; doi:10.1038/s41467-023-37277-3)
Supplement: Supplementary file 5 — Reporting Summary [file 41467_2023_37277_MOESM5_ESM.pdf]

nature portfolio

Corresponding author(s): Stefan N. Constantinescu

Last updated by author(s): 26-02-2023

Reporting Summary

Nature Portfolio wishes to improve the reproducibility of the work that we publish. This form provides structure for consistency and transparency in reporting. For further information on Nature Portfolio policies, see our Editorial Policies and the Editorial Policy Checklist.

Statistics

For all statistical analyses, confirm that the following items are present in the figure legend, table legend, main text, or Methods section.

n/a	Confirmed	
☐	☒	The exact sample size (n) for each experimental group/condition, given as a discrete number and unit of measurement
☐	☒	A statement on whether measurements were taken from distinct samples or whether the same sample was measured repeatedly
☐	☒	The statistical test(s) used AND whether they are one- or two-sided<br>*Only common tests should be described solely by name; describe more complex techniques in the Methods section.*
☐	☒	A description of all covariates tested
☐	☒	A description of any assumptions or corrections, such as tests of normality and adjustment for multiple comparisons
☐	☒	A full description of the statistical parameters including central tendency (e.g. means) or other basic estimates (e.g. regression coefficient) AND variation (e.g. standard deviation) or associated estimates of uncertainty (e.g. confidence intervals)
☐	☒	For null hypothesis testing, the test statistic (e.g. F, t, r) with confidence intervals, effect sizes, degrees of freedom and P value noted<br>*Give P values as exact values whenever suitable.*
☒	☐	For Bayesian analysis, information on the choice of priors and Markov chain Monte Carlo settings
☒	☐	For hierarchical and complex designs, identification of the appropriate level for tests and full reporting of outcomes
☒	☐	Estimates of effect sizes (e.g. Cohen's d, Pearson's r), indicating how they were calculated

Our web collection on statistics for biologists contains articles on many of the points above.

Software and code

Policy information about availability of computer code

Data collection	Peptides from HDx-MS spectrum were identified with the PLGS3.0 software (Waters).
Data analysis	The peptides identified by PLGS3.0 software (Waters) were analyzed with DynamX 3.0 (Waters). Data was statistically analyzed using Deuteros 2.0 with peptide-level significance testing (Lau et al., 2021).<br><br>Sequences of TpoR extracellular and transmembrane regions and of CALR Del52 were profiled for secondary structure, intrinsic disorder and accessibility propensities. Closest templates were retrieved with Phyre 2 50. Modeller (9.21) 51, Alpha-Fold2 (2.0) and Rosetta Folding (no versioning). For the study of interactions between CALR mutant tail and TpoR D1D2, docking trials were performed using three main start configurations of the complex based on the acidic areas of TpoR set as inputs in HADDOCK 2.4 for TpoR D1D2-CALR del52 mutant tail complex optimizations searches. The top configurations were further optimized using 500 ns Molecular Dynamics runs performed with OpenMM (7.4.1) 53 using a Monte Carlo Barostat, at 300K, using a Langevin integrator with 1ps-1 friction coefficient and a 2fs timestep and the FF14SB Force Field (Maier et al., 2014) to obtain 3 final poses in which the last residues of the mutant become unfolded. Free energy was estimated by both a knowledge-based method, using PRODIGY server (no versioning) and a physical MD estimation approach based on 3 simulations for each pose, using the MM-GBSA method at 150mM salt concentration, implemented in AMBER20. Conformational discretization for microstate analysis was performed using Time-Lagged Independent Component Analysis (TICA). The backbone dihedral angles of the CALR del52 mutant C-terminus molecule were used as input coordinates for TICA. TICA and free energy surfaces were computed using the PyEMMA (2.5.11) package, resulting plots were generated using the Matplotlib (3.5.1) package The inflection core state (InfleCS) clustering method was used to cluster the two transformed coordinates with the highest eigenvalues and the associated cluster centers were plotted on the corresponding free energy surface. Clustering was performed using 10 components, re-estimation of the same Gaussian mixture model was done 5 times.<br>Templates from AlphaFold2 and Rosetta Folding were used to effectively build the tetrameric 3D models and identify the interaction interface

between the two CALR mutants. HDx-MS data was used to identify contacts between TpoR and CALR Del52 in the formation of the tetramer complex. The ER specific G1M9 glycans of TpoR in contact to CALR were modeled with Glycopack (Westerlund AM, Delemotte L., 2019) in the configuration consistent with NMR data while the rest are of complex type, built in agreement with SAGS Database (https://sags.biochim.ro/) . The HDx-MS identified contacts and the solid-NMR data on the TM region configuration of TpoR dimer were used as constraints in generating the overall 2CALR-2TpoR model. This glycoproteic tetramer was immersed into a full-atom representation of the environment - consisting in a lipid bilayer of 1907 POPC molecules accommodating the TM region of TpoR and in 478479 TIP3P water molecules, 1328 chloride and 1402 sodium ions describing the solvent region hydrating the rest of the tetramer using the CHARMM-GUI server. This overall system consisting of ~ 1 million atoms was subjected to a mild simulated annealing procedure consisting in a start minimization, heating to 300K followed by cooling to 0K and final extended minimization, using NAMD (2.13) CHARMM36 forcefield. The same procedure was used to build TpoR-CALR Ins5 complex. The TpoR-CALR-Del52/ins5 models were further subjected to 3 molecular dynamics runs to explore the configuration sample space. More detailed protocols, including intermediate modelling steps, free energy estimates and detailed TICA analysis are presented in Supplementary Information.

For manuscripts utilizing custom algorithms or software that are central to the research but not yet described in published literature, software must be made available to editors and reviewers. We strongly encourage code deposition in a community repository (e.g. GitHub). See the Nature Portfolio guidelines for submitting code & software for further information.

Data

Policy information about availability of data

All manuscripts must include a data availability statement. This statement should provide the following information, where applicable:

- Accession codes, unique identifiers, or web links for publicly available datasets
- A description of any restrictions on data availability
- For clinical datasets or third party data, please ensure that the statement adheres to our policy

Mass spectrometry data was deposited on the ProteomeXchange repository (https://www.proteomexchange.org) under accession number PXD034131. PDB files of the molecular dynamics simulations were deposited on Figshare (link: https://figshare.com/s/b4ceb87fdce1f242e469 and https://figshare.com/s/9033970b5a1d3f8d6fa7.The source data file provides the raw data and reporting of HDx-MS experiments following the guidelines suggested by Masson et al.. Source data of other experiments are also provided with this paper in the source data file.

The ER specific G1M9 glycans of TpoR in contact to CALR were modeled with Glycopack (Westerlund AM, Delemotte L., 2019) in the configuration consistent with NMR data while the rest are of complex type, built in agreement with SAGS Database available at https://sags.biochim.ro/.

Human research participants

Policy information about studies involving human research participants and Sex and Gender in Research.

Reporting on sex and gender	NA
Population characteristics	NA
Recruitment	NA
Ethics oversight	NA

Note that full information on the approval of the study protocol must also be provided in the manuscript.

Field-specific reporting

Please select the one below that is the best fit for your research. If you are not sure, read the appropriate sections before making your selection.

[x] Life sciences [ ] Behavioural & social sciences [ ] Ecological, evolutionary & environmental sciences

For a reference copy of the document with all sections, see nature.com/documents/nr-reporting-summary-flat.pdf

Life sciences study design

All studies must disclose on these points even when the disclosure is negative.

Sample size

For in vitro experiments, no statistical method was used to predetermine sample size. Sample size was determined based on number of required to achieve statistical significance using non-parametric tests and on best practice and commonly used sample size in the field (e.g. Pecquet et al., Blood Jun 20;133(25):2669-2681).
For HDx-MS experiments, sample size was based on recommendations described in Masson et al., Nat Methods 16; 595-602 (2019). The quality (proper folding, purity) of purified proteins used for HDx-MS experiments was determined by microscale thermophoresis and

	Coomassie blue staining prior to the experiments. Three independent labeling reactions were performed for each time point for each conditions as described in methods. These reactions were randomized.<br>For in vitro functional and biochemical experiments, at least 2 and usually 3 or more independent biological replicates were performed for all experiments, as described in figure legends and in methods.
Data exclusions	No data were excluded from this study.
Replication	All experiments were repeated as indicated in the figure legends. All attempts at replication were successful. The number of biological replicates (n) is indicated in figure legends.
Randomization	Randomization was used for HDx-MS experiments following recommendation from Masson et al., Nat Methods 16; 595-602 (2019). Randomization was not relevant for in vitro studies beyond HDx-MS data generation since all experiments were performed identically with scientific method and in multiple replicates as indicated in figure legends.
Blinding	Blinding was not relevant for HDx-MS data generation as these were generated using an automated robotic system. For in vitro studies, blinding was not possible as the experimental conditions needed to be known by the researcher. All conditions were processed identically and with scientific method to avoid bias.

Reporting for specific materials, systems and methods

We require information from authors about some types of materials, experimental systems and methods used in many studies. Here, indicate whether each material, system or method listed is relevant to your study. If you are not sure if a list item applies to your research, read the appropriate section before selecting a response.

Materials & experimental systems

n/a	Involved in the study
☐	☒ Antibodies
☐	☒ Eukaryotic cell lines
☒	☐ Palaeontology and archaeology
☒	☐ Animals and other organisms
☒	☐ Clinical data
☒	☐ Dual use research of concern

Methods

n/a	Involved in the study
☒	☐ ChIP-seq
☒	☐ Flow cytometry
☒	☐ MRI-based neuroimaging

Antibodies

Antibodies used	Antibodies for used for co-immunoprecipitation:<br><br>Anti-FLAG tag (Genscript, Cat. No. A00184)<br>Mouse IgG control (Genscript, Cat. No. A01730)<br><br>Antibodies for western blot:<br><br>Anti-HA tag (Cell Signaling, clone C29F4 mAB #3724)<br>Anti-CALR mutant (Myelopro, clone SAT602)<br>Anti-rabbit IgG, HRP-linked (Cell Signaling, #7074)<br>Anti-FLAG tag (Genscript, Cat. No. A00184)<br>Anti-mouse IgG, HRP-linked (Cell Signaling, #7076)
Validation	Validation for antibodies used for co-immunoprecipitation:<br><br>Anti-FLAG tag (Genscript, Cat. No. A00184): validating by the manufacturer (Genscript) for immunoprecipitation from whole cell lysates and in this manuscript using (1) IgG control for immunoprecipitation and (2) cells expressing the co-immunoprecipitation partner but not the flag-tagged construct (Figure 2d, 3a, 5b).<br><br>Mouse IgG control (Genscript, Cat. No. A01730): control IgG used in Figure 2d, 3a,5b. Mouse IgG purified and validated by the manufacturer for purity.<br><br>Antibodies for western blot:<br><br>Anti-HA tag (Cell Signaling, clone C29F4 mAB #3724): validated by the manufacturer (Cell Signaling) on transfected cells expressing HA-tagged construct at the dilution used in this study (1:1000). The antibody was also validated in all experiments using a negative control of cells non transfected with HA-tagged proteins.<br><br>Anti-CALR mutant (Myelopro, clone SAT602): the antibody was validated for specificity for the mutant CALR against CALR WT and other proteins in Pecquet et al. Blood 2019, Pecquet, Papadopoulos et al., Blood 2022. It was further validated in this manuscript by using cells expressing CALR WT but not mutant CALR in Figure 2d, 3a.

Anti-rabbit IgG, HRP-linked (Cell Signaling, #7074): validated by the manufacturer to react (only) with rabbit antibodies by western blot.

Anti-FLAG tag (Genscript, Cat. No. A00184): Anti-FLAG tag (Genscript, Cat. No. A00184): validating by the manufacturer (Genscript) for western blot n this manuscript using cells expressing not expressing the flag-tagged construct.

Anti-mouse IgG, HRP-linked (Cell Signaling, #7076): validated by the manufacturer to react (only) with mouse antibodies by western blot.

Eukaryotic cell lines

Policy information about cell lines and Sex and Gender in Research

Cell line source(s)	HEK293T were from the American Type Culture Collection (ATCC). The Ba/F3 cell line (IL-3 dependent pro-B cells) were initially obtained after isolation of clones with pro-B lymphocytes characteristics (immune profiling and capacity to differentiate into B cells) from bone marrow of BALB/c mice as described in Palacios and M. Steinmetz, Cell, Vol. 41, 1985, pp.727-734. The cells were transferred from the laboratory of Prof. Merton Bernfield, Harvard Medical School, Children's Hospital, Boston MA to the laboratory of Prof. Harvey Lodish at Whitehead Institute, MIT, Cambridge MA in 1997. The cells were then transferred to Ludwig Institute for Cancer Research, Brussels Branch in 2001.
Authentication	HEK293T, ATCC were identified by STR profiling by the supplier (ATCC). Ba/F3 cell lines were identified by morphology, doubling time and dependence on IL-3.
Mycoplasma contamination	All cell lines used in this study were negative for mycoplasma and regularly tested for mycoplasma contamination by polymerase chain reaction (PCR).
Commonly misidentified lines (See ICLAC register)	No known misidentified cell line was used in this study.
